# The Effect of Growth Factors on Vaginal Wound Healing: A Systematic Review and Meta-analysis

**DOI:** 10.1089/ten.teb.2022.0225

**Published:** 2023-08-08

**Authors:** Melissa J.J. van Velthoven, Aksel N. Gudde, Frederique Struijs, Egbert Oosterwijk, Jan-Paul Roovers, Zeliha Guler, Carlijn R. Hooijmans, Paul H.J. Kouwer

**Affiliations:** ^1^ Institute of Molecules and Materials, Radboud University, Nijmegen, The Netherlands.; ^2^ Department of Urology, Radboud Institute for Molecular Life Sciences, Radboud University Medical Center, Nijmegen, The Netherlands.; ^3^ Department of Obstetrics and Gynecology and Amsterdam University Medical Center, Amsterdam, The Netherlands.; ^4^ Amsterdam Reproduction and Development, Amsterdam University Medical Center, Amsterdam, The Netherlands.; ^5^ Department of Anesthesiology, Pain and Palliative Care, Radboud University Medical Center, Nijmegen, The Netherlands.

**Keywords:** wound healing, pelvic organ prolapse, growth factors, basic fibroblast growth factor, connective tissue growth factor, epidermal growth factor

## Abstract

Surgical outcomes of pelvic organ prolapse (POP) surgery are poor, resulting in a 20% recurrence risk. Following the hypothesis that impaired wound healing is the main determinant of recurrent POP, growth factors have the potential to promote wound healing and may improve surgical outcomes. In this study, we systematically reviewed the effect of growth factors on vaginal wound healing in both *in vitro* and animal studies. For each independent comparison, the standardized mean difference and 95% CI were calculated using the Hedges' g correction. Of the 3858 retrieved studies, seven studies were included, of which six were included in meta-analysis (three *in vitro* studies and four *in vivo* studies). *In vitro*, basic fibroblast growth factor (bFGF) promotes proliferation, differentiation, and collagen types I and III production. Epidermal growth factor stimulates proliferation and connective tissue growth factor promotes Tenascin-C expression. These effects, however, are less pronounced *in vivo*; only bFGF slightly promotes collagen production. The review shows that growth factors, particularly bFGF, are able to promote vaginal wound healing *in vitro*. The uncertain *in vivo* findings suggest that preclinical models should be improved. The ultimate goal is to develop effective growth factor-supplemented therapies that improve surgical outcomes for POP.

**Impact statement:**

Growth factors, particularly basic fibroblast growth factor, promote vaginal wound healing *in vitro*, but the effects are less prominent *in vivo*. To improve surgical outcomes for pelvic organ prolapse in the future, more research is required, including improved preclinical models.

## Introduction

The lifetime risk for pelvic organ prolapse (POP) surgery is 12.6%,^1^ corresponding to 166,000 operations in 2010 in the United States, which may increase to 310,050 in 2050.^2^ POP is characterized by the descent of pelvic organs into the vagina, due to the weakening of the pelvic floor supportive tissues. Surgical outcomes of POP are poor with ∼20% recurrence after native tissue repair.^3^ Compared to healthy tissues, POP tissues are characterized by an increased fibroblast contractility and an altered extracellular matrix (ECM) composition (growth factor densities and collagen content), resulting in disrupted tissue integrity and reduced load-bearing capabilities.^4,5^

Reciprocally, the disrupted tissue integrity has impairing effects on the subsequent wound healing processes. It has been hypothesized that the high recurrence rate originates from impaired wound healing, possibly through the altered functionality of POP fibroblasts.^5^ To improve surgical outcomes, implants such as polypropylene meshes have been introduced with frequent severe adverse events after implantation.^6^ Currently, there is an urgent need for developing alternative strategies to promote vaginal wound healing to improve surgical outcomes for POP.

A promising strategy is tissue engineering, which uses the combination of cells, biomaterials, and biomechanical and biochemical cues to regenerate tissues.^6^ Multiple cell types have been investigated regarding pelvic tissue regeneration, such as fibroblasts,^7^ bone marrow-derived stem cells,^8^ adipose-derived stem cells (ADSCs),^9^ endometrial mesenchymal stem cells (MSCs),^10,11^ and skeletal muscle-derived stem cells.^12^ Biomaterials such as polycaprolactone,^13^ polylactic acid,^14,15^ and polylactic-co-glycolic acid^9^ have shown promise in triggering a regenerative pelvic tissue response.

Moreover, biomechanical cues are capable of stimulating fascia tissue regeneration.^16^ As for biochemical cues, a recent systematic review details that estrogen improves vaginal wound closure, collagen production, and tissue strength *in vivo*.^17^ Also, studies with growth factors have shown promising results; their signaling pathways are generally well understood as is their role in wound healing.^5,18^ Their true impact on stimulating vaginal wound healing, however, is still relatively unclear.^5,19,20^ Although systematic reviews have examined the potential of growth factors in bone regeneration,^21,22^ liver regeneration,^23^ and periodontal regeneration,^24^ no systematic review has been published covering the effect of growth factors in the context of vaginal wound healing.

In this study, we examine the effect of growth factors on vaginal wound healing by providing a systematic overview of both *in vitro* and animal studies. The former was included to provide a comprehensive overview of the field. Through conducting a meta-analysis, we quantitatively assess the efficacy of each growth factor on outcome measures describing vaginal wound healing. This systematic review improves understanding of the effect of these growth factors and suggests directions on how to improve translatability of *in vitro* results, which ultimately should aid the surgical outcomes for POP.

## Method

To study the effect of growth factors on vaginal wound healing, this systematic review was reported according to the Preferred Reporting Items for Systematic Reviews and Meta-Analysis (PRISMA) guidelines.^25^ The protocol was registered in the international prospective register of systematic review (PROSPERO) on January 15th, 2021 (CRD42021226841). An amendment to the protocol was made on May 21st, 2021, whereby an additional inclusion criterion during the abstract screening was added: outcomes describing vaginal wound healing.

### Literature search

The systematic literature search was conducted in Ovid MEDLINE, through the PubMed interface, and Ovid EMBASE to identify all relevant studies up to March 22nd, 2021. The SYRCLE step-by-step guide was used to design a comprehensive search strategy.^26^ The search strategy consisted of MeSH and EMTREE terms for PubMed and EMBASE, respectively, as well as title/abstract (tiab) terms for all three search domains: (1) growth factors; (2) vaginal wound healing, POP, pelvic floor, or vaginal reconstruction/repair; and (3) *in vitro* and animal filter (for complete search strategy, see Supplementary Tables S1 and S2). No additional restriction was applied. Cited articles of included studies and relevant reviews were crosschecked for potentially relevant studies. The studies were imported and de-duplicated in EndNote X9 (Clarivate Analytics).

### Study selection

The studies were independently screened by two reviewers (M.J.J.v.V. and A.N.G.). The initial round of screening was based on title and abstract, which was conducted using the Rayyan web tool.^27^ The following inclusion criteria were used: (1) an original research article, (2) a preclinical (animal/*in vitro*) study, (3) growth factor administration/supplementation, and (4) outcome measures describing vaginal wound healing. In the second round of screening, full texts were screened using the same exclusion criteria as mentioned above, including an additional fifth inclusion criterion: in the case of animal studies, growth factor(s) should be locally delivered. Discrepancies between the reviewers were resolved through discussion between the two reviewers, and when no consensus was reached, a third reviewer (Z.G.) was consulted. At last, screening was performed based on reference lists of included studies and relevant reviews to check for potentially missing studies.

### Study characteristic extraction

The following study design characteristics were extracted from all included studies: bibliographical data (author and year), experimental and control groups, time point outcome assessment, type of growth factor, growth factor administration method, dosage, and reported outcome measures (both descriptive and quantitative data) describing vaginal wound healing. For *in vitro* studies, the following *in vitro* model details were reported: cell type, passage number, healthy or diseased, and premenopausal or postmenopausal status. For *in vivo* studies, the following animal model characteristics were reported: species, strain, healthy or diseased, premenopausal or postmenopausal, parous state, location of intervention, and survival. Authors were contacted in the case of missing study characteristics.

### Quality assessment

For the *in vitro* studies, quality assessment was performed using an adapted version of the commonly used OHAT tool (Supplementary Table S3).^28^ Risk of bias was assessed for all included animal studies using the SYRCLE Risk of Bias tool for animal studies.^29^ Since reporting of experimental details on animals, methods, and materials is generally very poor,^30,31^ we added two items on reporting to overcome the problem of judging too many items as unclear risk of bias: “reporting of any measure of randomization” and “reporting of any measure of blinding.” Two independent reviewers assessed the risk of bias in the included studies (M.J.J.v.V. and F.S.). Discrepancies were solved through discussion, and when no consensus was reached, the methodologist (C.R.H.) was consulted.

### Meta-analysis

The mean, variability and uncertainty measures (standard deviation [SD], standard error of the mean [SEM] or 95% confidence interval [95% CI]), and sample size (*n*) were extracted for each independent comparison of all quantitative reported outcome measures described in Supplementary Tables S4 and S5. Data applicable for meta-analysis were extracted independently by two reviewers (M.J.J.v.V. and A.N.G.). The first reviewer (M.J.J.v.V.) checked the data for discrepancies and the data of M.J.J.v.V. were used for the meta-analysis. When the data were only visualized in the figures, a digital ruler software was used (FIJI). Authors were contacted through e-mail in the case of incomplete data.

Meta-analysis was performed with Comprehensive Meta-Analysis (CMA; version 3.0) only when a minimum of four independent comparisons was reached for each reported outcome, per growth factor type, and separately for *in vitro* and *in vivo* studies. When the mean, variability and uncertainty measures, or sample size could not be extracted, the comparison was excluded for meta-analysis.

In case sample sizes were provided in a range or there was a mismatch between methods and results, the lowest number was used for meta-analysis. When it was unknown whether the SD or SEM was reported, we assumed SEM to prevent overestimation of the variability and uncertainty measures. For the meta-analysis, the SEM and 95% CI were translated to SD using the following formulas: (1) SD=SEM∗√n and (2) $SD=\surd n*\left(upper-lowerlimit\right)∕3.92$.^32^ A correction was performed for multiple use of the control group by adjusting the sample size of the control group (sample size of control group divided by the amount the control group was used) to a minimum of two.

For each independent comparison, the standardized mean difference (SMD) and 95% CI were calculated using the Hedges' g correction. When similar outcomes were reported in the same study, priority was given to the primary outcome described in the PROSPERO protocol. For *in vitro* studies, we assumed that in the case of outcome assessment on multiple time points, independent experiments were conducted. The random-effects model was used to calculate the overall effect, which assumes that the observed treatment effect can vary between studies due to differences in treatment effect in each study as well as sampling variability.^33^ The statistical method *I*^2^ was used to assess heterogeneity.

For the following predefined study, characteristic subgroup analysis was conducted in the case of at least five independent comparisons in a minimum of three studies: species, administration route, type of surgical procedure, dosage, and time point outcome assessment. For time point outcome assessment, we categorized “short” as the first 7 days, “middle” as 8–14 days, and “long” as more than 14 days.

### Sensitivity and publication bias analyses

Sensitivity analyses were conducted in the case of at least four comparisons to examine the robustness of the main analyses. First, we investigated the effect of a possible interaction based on time point outcome assessment for the *in vitro* outcomes. We checked whether the assumption that all reported time points were independent comparisons showed similar results compared to the assumption that multiple time points are repeated measures. Second, we investigated the effect of a possible interaction based on different methods reporting the same outcome, by performing sensitivity analyses for each method independently. For the assessment of publication bias, funnel plots were generated in the case of a minimum of 15 studies of a specific outcome.

## Experiment

### Study selection

**FIG. 1.**
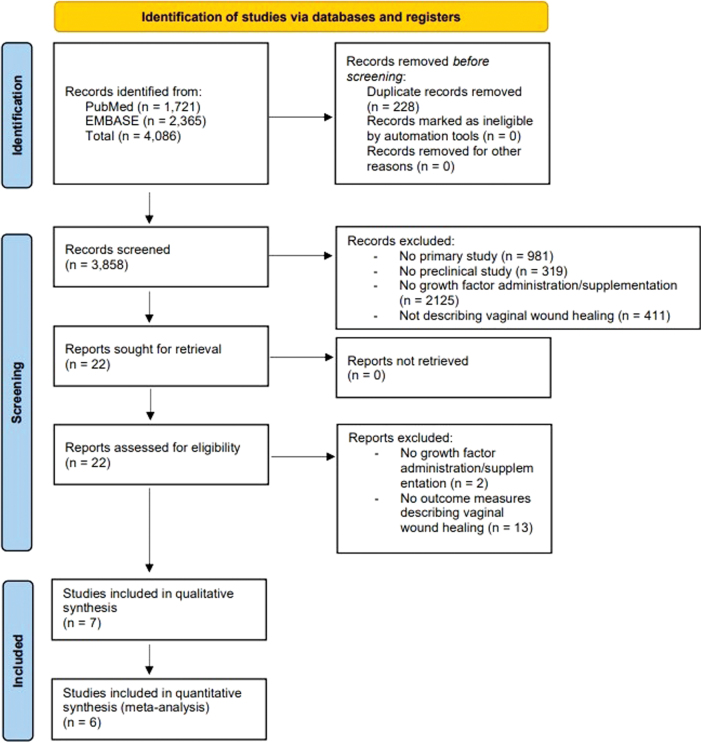
PRISMA flow diagram. Color images are available online.

The comprehensive literature search resulted in 3858 unique records, which were assessed for eligibility (Fig. 1). Twenty-two studies met our inclusion criteria based on title and abstract screening. After full-text screening, seven studies were included in this systematic review, of which six studies could be included in the meta-analysis.

### Study characteristics

The study characteristics of the included *in vitro* and *in vivo* studies are summarized in Table 1 (Supplementary Tables S4 and S5 for detailed version). Of Jin et al., only the *in vitro* section was included in the systematic review as the *in vivo* experiments did not include an appropriate control.^8^ Basic fibroblast growth factor (bFGF) is the most reported growth factor (six studies), followed by connective tissue growth factor (CTGF) (two studies) and epidermal growth factor (EGF) (one study).

**Table 1.** Study Characteristics of All Included Studies

Included in vitro studies						
Study	Cell type	Type of GF	Administration method	Dosage	Time point outcome assessment	Reported outcomes
Jia et al. (2018)^7^	ADSCs (rat)	bFGF	Soluble	1, 10 and 100 ng/mL	Day 1–7	Proliferation
		EGF	Soluble	1, 10 and 100 ng/mL	Day 1–7	Proliferation
Jin et al. (2016)^8^	Elastin-transfected BMSCs (rat)	bFGF	Loaded in PLGA nanoparticles	20 ng/mL	Day 0–7	Proliferation
Su et al. (2014)^46^	eMSCs (human)	CTGF	Soluble	10, 50 and 100 ng/mL	Day 14 and 28	Differentiation into fibroblasts,^a^ protein expression of collagen type I and Tenascin-C
		CTGF	Soluble^b^	100 ng/mL	Day 14 and 28	Protein expression of collagen type I and Tenascin-C
Wu et al. (2022)^61^	ADSCs (rat)	bFGF	Transfection	MOI of 100	Day 0–7, day 14 and day 28	Proliferation, differentiation into fibroblasts, expression of ECM (collagen type I and III) and inflammatory (IL-1β, TNF-α and IL-6) proteins
		bFGF	Transfection^c^	MOI of 100	Day 0–6	Proliferation
		bFGF	Transfection^d^	MOI of 100	Day 0, 1, 3, 5, 7, 9 and 11	Proliferation, protein expression collagen type I and III, HUVEC tube formation assay
		bFGF	Soluble	20 ng/mL	Day 14 and 28	Differentiation into fibroblasts, protein expression collagen type I and III
Zhang et al. (2016)^62^	Fibroblast (human)	bFGF	Mesh	NR^e^	Day 1, 3 and 5	Cell viability^a^ and proliferation

Included in vivo studies						
Study	Species (strain)	Type of GF	Administration method (location)	Dosage	Time point outcome assessment	Reported outcomes
Glindtvad et al. (2018)^63^	Rats (Wistar)	bFGF	Mesh (abdominal wall)	0.35 μg/mesh	Week 4, 8 and 24	Macroscopic evaluation, biomechanical properties, histology^a^ (collagen), *COL1A1*, *COL3A1* and *FN1* expression, protein expression of collagen type I and III and total collagen content
Hansen et al. (2020)^64^	Rats (Wistar)	bFGF	Mesh (abdominal wall)	10 μg/mesh	Week 8 and 24	Macroscopic evaluation, biomechanical properties, histology (inflammatory response and collagen), *COL1A1* and *COL3A1* expression, protein expression of collagen type I and III and total collagen content
		CTGF	Mesh (abdominal wall)	9 μg/mesh	Week 8 and 24	
Zhang et al. (2016)^62^	Rabbits (New Zealand White)	bFGF	Mesh (abdominal wall)	NR^e^	Week 4, 8 and 12	Biomechanical properties, histology (inflammatory response^a^ and collagen), protein expression of collagen

^a^ Descriptive outcome measure.

^b^ eMSCs cultured on a polyamide mesh.

^c^ Co-cultured with fibroblasts.

^d^ Conditioned medium of bFGF-transfected ADSCs cultured on HUVECs.

^e^ 10 mg/mL bFGF in 10 mM Tris, pH 8.5.

ADSC, adipose-derived stem cell; BMSC, bone marrow-derived stem cell; eMSC, endometrial mesenchymal stem cell; GF, growth factor; bFGF, basic fibroblast growth factor; EGF, epidermal growth factor; CTGF, connective tissue growth factor; PLGA, poly(lactic-co-glycolic) acid; MOI, multiplicity of infection; NR, not reported; ECM, extracellular matrix; IL-1β, interleukin-1β; TNF-α, tumor necrosis factor α; IL-6, interleukin-6; HUVEC, human umbilical vein endothelial cell.

Regarding *in vitro* studies, dosage and type of administration varied across the studies, such as soluble administration (three studies), loaded in nanoparticles, incorporated into a polypropylene mesh, and transfected (each one study). The main reported *in vitro* outcomes related to vaginal wound healing are proliferation (four studies), (stem)cell differentiation into fibroblasts (two studies), and protein expression of collagen type I (two studies), type III (one study), and Tenascin-C (TNC) (one study).

Regarding *in vivo* studies, bFGF is the most reported growth factor (three studies). In addition, one study also investigated the effect of CTGF on vaginal wound healing. The administration method and location of implantation are similar across all *in vivo* studies: incorporated into a mesh implanted in the abdominal wall (three studies). In this study, the main reported outcomes related to vaginal wound healing are macroscopic evaluation (two studies), biomechanical properties (three studies), histological assessment focusing on the inflammatory response (two studies) and collagen expression (three studies), gene and protein expression of collagen type I and type III (two studies), and total collagen measurements (three studies).

### Quality assessment

To assess the quality of included studies, we assessed risk of bias in all studies. In 33% of the *in vivo* studies, authors reported randomization of animals across various groups. In contrast, in none of the *in vitro* studies, authors reported any randomization protocol during the conduct of their experiments (Supplementary Fig. S1). Randomization was not performed in any of the included studies regarding random selection during outcome assessment. Blinding at any level was reported in 66% of the *in vivo* studies and not reported in the *in vitro* studies. Sixty-six percent of the *in vivo* studies inadequately addressed incomplete outcome data, resulting in a high risk of bias. Many risk of bias domains scored an unclear risk of bias, which indicates that methodological details of included studies were often incomplete. For example, in none of the *in vivo* and *in vitro* studies, the allocation sequence was described.

### Meta-analysis

As not always all outcome data could be retrieved, only six studies were included in the meta-analysis (Fig. 1). Jin et al. was excluded from the meta-analysis, as the sample size of the reported outcome could not be retrieved. The meta-analysis consists of both *in vitro* and *in vivo* analyses. In the *in vitro* analyses, we assessed the effect of growth factors on proliferation, differentiation into fibroblasts, and the expression of ECM proteins collagen type I and III and TNC. In the *in vivo* analyses, we determined the effect of growth factors on collagen expression and biomechanical properties.

#### *In vitro* analyses

##### Proliferation

**FIG. 2.**
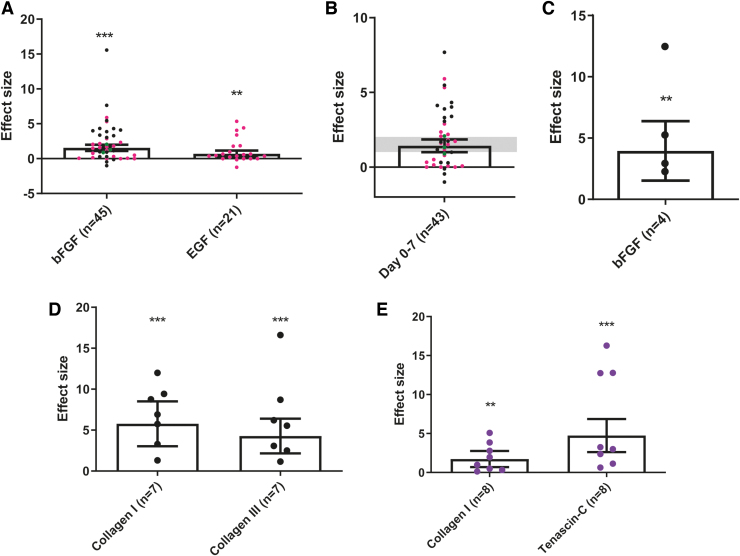
Overview of the meta-analysis results of the effect of growth factors on proliferation **(A, B)**, (stem)cell differentiation into fibroblasts, **(C)** and ECM protein expression **(D, E)** *in vitro*. **(A)** bFGF (SMD = 1.54 [1.10–1.98]) and EGF (SMD = 0.69 [0.23–1.15]) stimulate proliferation. **(B)** Subgroup analysis, looking at short-term outcome assessment, supports that bFGF promotes proliferation (SMD = 1.43 [1.00–1.86]) **(***gray bar* illustrates the 95% CI of the main analysis shown in **A)**. **(C)** bFGF has a stimulatory effect on (stem)cell differentiation into fibroblasts (SMD = 3.96 [1.54–6.39]). **(D)** bFGF promotes the protein expression of both collagen type I (SMD = 5.77 [3.03–8.52]) and collagen type III (SMD = 4.27 [2.15–6.39]). **(E)** CTGF increases collagen type I (SMD = 1.72 [0.69–2.76]) and Tenascin-C (SMD = 4.74 [2.61–6.86]) expression on protein level. Bar charts represent the SMD ±95% CI (**p < 0.01 and ***p < 0.001). The individual data points correspond to the following studies: Jia et al. in *pink*, Su et al. in *purple*, Wu et al. in *black* and Zhang et al. in *green*. ECM, extracellular matrix; bFGF, basic fibroblast growth factor; CTGF, connective tissue growth factor; SMD, standardized mean difference. Color images are available online.

bFGF significantly stimulates proliferation (SMD = 1.54, 95% CI = [1.10–1.98], *n* = 45, *I*^2^ = 69%, *p* < 0.001) (Fig. 2A and Supplementary Fig. S2). To determine the effect of bFGF on proliferation at different time points, subgroup analysis was performed based on time point outcome assessment. Regarding short term, the effect size is similar to the main analysis (SMD = 1.43, 95% CI = [1.00–1.86], *n* = 43, *I*^2^ = 65%) (Fig. 2B and Supplementary Fig. S3). We could not assess the effect on proliferation at middle or long term, due to the limited number of independent comparisons reporting at later time points. EGF also promotes proliferation (SMD = 0.69, 95% CI = [0.23–1.15], *n* = 21, *I*^2^ = 48%, *p* = 0.004), but the effect size is lower in comparison to bFGF (Fig. 2A and Supplementary Fig. S4).

##### (Stem) cell differentiation into fibroblasts

Meta-analysis revealed that treatment with bFGF significantly promotes ADSC to fibroblast differentiation based on histological expression of fibroblast-specific protein 1 (FSP-1) (SMD = 3.96, 95% CI = [1.54–6.39], *n* = 4, *I*^2^ = 61%, *p* = 0.001) (Fig. 2C and Supplementary Fig. S5).

##### ECM metabolism

bFGF significantly upregulates collagen type I (SMD = 5.77, 95% CI = [3.03–8.52], *n* = 7, *I*^2^ = 75%, *p* < 0.001) and collagen type III (SMD = 4.27, 95% CI = [2.15–6.39], *n* = 7, *I*^2^ = 71%, *p* < 0.001) (Fig. 2D and Supplementary Fig. S6). CTGF significantly increases the expression of TNC (SMD = 4.74, 95% CI = [2.61–6.86], *n* = 8, *I*^2^ = 88%, *p* < 0.001) and collagen type I (SMD = 1.72, 95% CI = [0.69–2.76], *n* = 8, *I*^2^ = 76%, *p* = 0.001) (Fig. 2E and Supplementary Fig. S7A, B).

#### *In vivo* analyses

##### Collagen expression

**FIG. 3.**
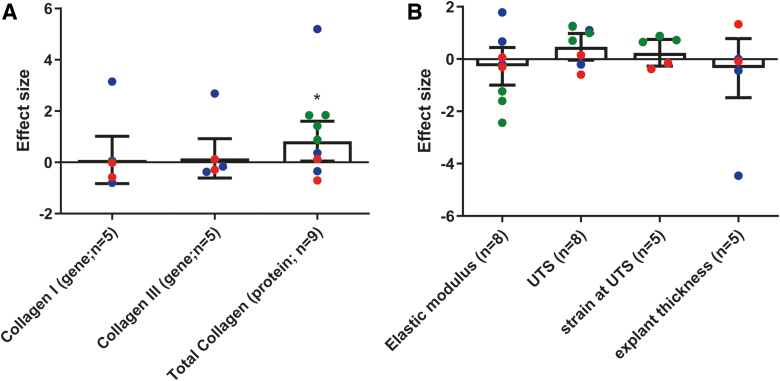
Overview of the meta-analysis results of the effect of growth factors on collagen expression **(A)** and biomechanical properties **(B)** *in vivo*. **(A)** bFGF treatment stimulates total collagen production (SMD = 0.83 [0.05–1.60]), but it has no effect on *COL1A1* (SMD values) and *COL3A1* expression (SMD values) (SMD = 0.16 [−0.61 to 0.92]). **(B)** bFGF has no effect on the following biomechanical properties: elastic modulus (SMD = −0.28 [−1.00 to 0.45]), UTS (SMD = 0.47 [−0.04 to 0.98]), strain at UTS (SMD = 0.24 [−0.27 to 0.75]) and explant thickness (SMD = −0.35 [−1.47 to 0.78]). Bar charts represent the SMD ±95% CI (*p < 0.05). The individual data points correspond to the following studies: Glindtvad et al. in *blue*, Hansen et al. in *red* and Zhang et al. in *green*. Color images are available online.

No effect of bFGF on *COL1A1* (SMD values) and *COL3A1* expression was observed (SMD values) (SMD = 0.16, 95% CI = [−0.61 to 0.92], *n* = 5, *I*^2^ = 59%). For total collagen production, bFGF has a slight promoting effect (SMD = 0.83, 95% CI = [0.05–1.60], *n* = 9, *I*^2^ = 71%, *p* = 0.036) (Fig. 3A and Supplementary Fig. S8A–C).

##### Biomechanical properties

bFGF has no effect on the biomechanical properties (Fig. 3B and Supplementary Fig. S9). The effect of bFGF was assessed for the following biomechanical properties: elastic modulus (SMD = −0.28, 95% CI = [−1.00 to 0.45], *n* = 8, *I*^2^ = 64%), ultimate tensile strength (UTS) (SMD = 0.47, 95% CI = [−0.04 to 0.98], *n* = 8, *I*^2^ = 37%), strain at UTS (SMD = 0.24, 95% CI = [−0.27 to 0.75], *n* = 5, *I*^2^ = 4%), and explant thickness (SMD = −0.35, 95% CI = [−1.47 to 0.78], *n* = 5, *I*^2^ = 79%).

#### Sensitivity and publication bias analyses

Sensitivity analyses were performed to test the robustness of our main analysis. First, when only the last reported time points were included instead of all reported time points, the promoting effect of CTGF on collagen type I *in vitro* appeared to be diminished (SMD = 1.87, 95% CI = [−0.04 to 3.77], *n* = 4, *I*^2^ = 85%), in contrast to a significant increase in the main analysis (Supplementary Fig. S7C). Second, on a histological level, bFGF does not affect collagen production *in vivo*, whereas in the main analysis, bFGF significantly increased collagen production (SMD = 0.56, 95% CI = [−0.32 to 1.44], *n* = 5, *I*^2^ = 60%) (Supplementary Fig. S8D). Publication bias could not be assessed due to the limited number of studies.

## Discussion

### Main findings

**FIG. 4.**
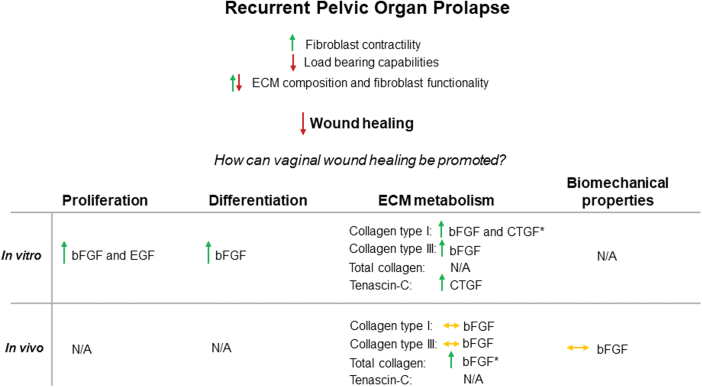
Schematic overview of the key findings. Impaired vaginal wound healing is a major determinant of recurrent POP. Growth factors have the potential of promoting vaginal wound healing regarding proliferation, (stem) cell differentiation into fibroblasts, ECM metabolism and biomechanical properties. The effects of the individual growth factors are summarized for *in vitro* and *in vivo* separately (*green* = promoting effect, *red* = impaired effect, *green*/*red* = altered effect, *orange* = no effect and N/A = not applicable). The *marks the effects that did not appear to be robust upon sensitivity analyses. POP, pelvic organ prolapse. Color images are available online.

The meta-analysis provides evidence, in the context of vaginal wound healing, that *in vitro* bFGF and EGF stimulate proliferation, bFGF promotes protein expression of collagen type I and III and differentiation into fibroblasts. In addition, CTGF stimulates the ECM protein expression of TNC *in vitro*. However, no promoting effect of bFGF on *COL1A1* and *COL3A1* expression, and biomechanical properties was observed *in vivo*. We note that bFGF significantly stimulates total collagen production, but this effect did not appear to be robust. A schematic overview of the key findings is presented in Figure 4.

### Strengths and limitations

Although it is the first systematic review describing the effect of growth factors on vaginal wound healing *in vitro* and *in vivo*, this review also has some limitations. First, the number of included studies in the meta-analysis is rather low, which may result in the imprecision of the overall effects, emphasizing that more research in the context of vaginal wound healing is required. Second, there was substantial heterogeneity between the included studies.

To account for the substantial heterogeneity between the included studies, we used the SMD and the random-effects model. Third, due to the limited number of included studies, we were not able to perform subgroup analyses for the following study characteristics: species, administration route, type of surgical procedure, duration, and dosage. In the case of treatment duration, we were able to perform subgroup analysis solely for the outcome proliferation upon bFGF treatment. Fourth, we were not able to assess the risk of publication bias due to a limited number of included studies.

We expect that our results are slightly overestimated as systematic reviews of preclinical studies have reported the presence of publication bias.^34^ Fifth, most of the included studies poorly reported all essential methodological details, which hampered the risk of bias assessment and possibly affected the reliability of our findings. Sixth, some potentially relevant outcomes could not be included due to a lack of methodological details or the use of proper control groups in multiple studies. Seventh, all *in vivo* studies used healthy premenopausal animals, whereas the majority of patients is postmenopausal. Postmenopausal status negatively affects vaginal wound healing due to reduced estrogen levels,^35^ making it more challenging to translate our findings to the clinical setting.

### Interpretation

#### Proliferation

Fibroblasts play a critical role in all three phases of wound healing as they deposit and remodel the ECM, and facilitate wound contraction. Fibroblast proliferation is crucial in (vaginal) wound healing and it is well established that cell proliferation is stimulated with growth factor treatment.^5^ Our meta-analysis indicates that bFGF and EGF promote proliferation *in vitro*. It has been reported that bFGF stimulates proliferation in the context of tendon^36^ and cartilage regeneration^37^ *in vitro*, and salivary gland regeneration *in vivo*^38^; and EGF in urothelial regeneration *in vivo*.^39^ So far, no *in vivo* research has been conducted regarding the effect of growth factors, such as bFGF, on proliferation during vaginal wound healing.

#### (Stem) cell differentiation into fibroblasts

During wound healing, MSCs become activated, are recruited to the wound, and differentiate into fibroblasts.^40^ For example, platelet-derived growth factor (PDGF)^41^ and vascular endothelial growth factor (VEGF)^42^ can promote wound healing in patients with diabetic foot ulcers, whereby PDGF and VEGF act as chemoattractants for MSCs.^43^ The meta-analysis revealed that bFGF significantly promotes ADSC differentiation into fibroblasts. However, the effect of growth factors on the recruitment and differentiation of (stem) cells into fibroblasts is still unknown for vaginal wound healing *in vivo*.

#### ECM metabolism

The relation between POP and connective tissue metabolism suggests that improved vaginal wound healing requires the regulation of ECM metabolism.^5,44^ The meta-analysis showed that bFGF stimulates collagen I and III production *in vitro*. CTGF also plays a key role in regulating ECM production. For example, an *in vitro* study demonstrated a promoting effect of CTGF on collagen deposition.^45^ Although our meta-analysis also showed that CTGF promotes the deposition of collagen type I *in vitro*, sensitivity analysis could not validate this effect.^46^ In line with our findings, Zhang et al. also demonstrated that *in vitro* CTGF treatment resulted in an upregulated expression of TNC in the context of renal tubulointerstitial fibrosis,^47^ emphasizing its important role in wound healing by upregulating collagen expression and maintaining the integrity of the ECM.^48^

Upon bFGF treatment, upregulation of collagen type I and III could not be observed *in vivo*. Moreover, the slight promoting effect of bFGF on total collagen content *in vivo* could not be validated by sensitivity analysis, which suggests that the effect of bFGF on collagen synthesis *in vivo* is still uncertain. In line with these findings, other *in vivo* studies presented contradictory results: both promoting^49^ and impairing^50^ effects of bFGF on collagen synthesis during wound healing were reported. A possible explanation for these contradictory results could be that collagen deposition during wound healing is intricately linked to fibrosis, which is caused by excessive collagen deposition in the case of impaired wound healing.^51^

Another explanation could be that the effect of growth factors on collagen expression is dependent on the dosage and duration of the growth factor treatment. However, this dependency could not be investigated through subgroup analysis, which prevented us from determining “optimal” growth factor concentrations. It should be noted that the optimal dose not only depends on the growth factor type but also varies according to the administration method and animal model.

#### Biomechanical properties

The pelvic floor is continuously exposed to mechanical loading; therefore, sufficient tissue strength is crucial for providing pelvic organ support. In POP patients, tissue integrity and strength^52^ of the pelvic floor are impaired, whereas the tissue of the (anterior) vaginal wall is stiffer compared to healthy tissue.^53^ This increased stiffness may contribute to POP as it can induce vaginal fibroblast to myofibroblast differentiation.^54^ Introducing a growth factor to the injured tissue could trigger a wound healing response characterized by tissue remodeling, which eventually affects the biomechanical properties. The meta-analysis showed that bFGF has no effect on the following biomechanical properties: elastic modulus (as an indication for tissue stiffness) and strain at UTS or UTS (indicators for tissue strength).

In the context of skin wound healing, Lu et al. demonstrated that bFGF administration resulted in an insignificant increase in tissue stiffness and a significant increase in tissue strength.^55^ However, data regarding POP-related biomechanical properties are limited in animal models.^56^ Whether POP biomechanics are adequately mimicked in these animal studies remains doubtful. Clearly, more research is required to determine the “optimal” vaginal tissue strength and stiffness in healthy tissue, as these are currently still unknown.

### Implications

Based on this systematic review, we conclude that growth factors have a promoting effect on vaginal wound healing *in vitro*. bFGF is the most promising candidate as it promotes proliferation, stem cell differentiation into fibroblasts, and collagen production. *In vivo*, bFGF promotes total collagen production as well, but this effect did not appear robust. This emphasizes that the effect of bFGF on collagen synthesis *in vivo* in the context of POP is still uncertain and that more research is required to determine the potential of bFGF in promoting collagen production, while avoiding fibrosis. In general, this discrepancy emphasizes that the effects observed *in vitro* do not translate to the (pre)clinical situation.

We suggest that the translation from *in vitro* to *in vivo* can be enhanced by developing more complex *in vitro* models, which better resemble the wound healing process of POP patients. In such a model, an organ-on-a-chip, for example, growth factors can be investigated under more relevant physiological conditions, which would reduce animal use in accordance with the 3Rs principle (replacement, reduction and refinement).^57^

To account for the fact that growth factor delivery is potentially suboptimal in promoting vaginal wound healing, these devices can be used to more accurately assess growth factor delivery and dosage in comparison to conventional *in vitro* assays. Furthermore, we advise that clinicians contribute to these *in vitro* models by defining which biological parameters should be included. We also suggest that future research should include other growth factors, such as VEGF, PDGF, and transforming growth factor β. By studying the effect of different growth factors in an *in vivo*-mimicking environment, translation to *in vivo* studies can be improved.

For the translation of animal studies to the clinic, it is crucial that the effect of growth factors on vaginal wound healing is investigated in validated vaginal (larger) animal models. We note that for this systematic review, only studies with healthy, abdominal rat and rabbit models were retrieved; these models provide an indication of growth factor effects on wound healing in general, but miss the vaginal context. We suggest that animal models with larger species are used for *in vivo* studies, such as sheep or nonhuman primates^58^ (e.g., rhesus macaques^59^ or squirrel monkeys^60^).

These animals can spontaneously develop POP and are therefore considered good models for POP. Furthermore, the anatomy of these animals better resembles the human vaginal architecture and physiology compared to the small animal models included in this review. As sheep are more ethically acceptable, we advise that future *in vivo* studies that examine the effect of growth factors on vaginal wound healing are performed in an ovine vaginal model. We also advise that during animal studies, the delivery and dosage of the growth factor are critically evaluated.

Growth factors are able to promote vaginal wound healing *in vitro*. Improved *in vitro* models as well as validated larger animal models can stimulate the translation from these preclinical findings to the clinic and thus the development of effective growth factor-supplemented therapies. In the future, these therapies have the potential to improve the surgical outcomes for POP, resulting in a lower recurrence rate in patients.

## Authors' Contributions

M.J.J. v.V.: conceptualization, investigation, data curation, formal analysis, funding acquisition, writing – original draft, and writing – review and editing. A.N.G.: conceptualization, investigation, data curation, and writing – review and editing. F.S.: investigation and writing – review and editing. E.O.: supervision and writing – review and editing. J.P.R.: conceptualization, supervision, funding acquisition, and writing – review and editing. Z.G.: conceptualization, supervision, funding acquisition, and writing – review and editing. C.R.H.: conceptualization, investigation, methodology, supervision, and writing – review and editing. P.H.J.K.: conceptualization, supervision, funding acquisition, and writing – review and editing. All authors read and approved the final article.

## Supplementary Material

Supplemental data

Supplemental data

Supplemental data

Supplemental data

Supplemental data

Supplemental data

Supplemental data

Supplemental data

Supplemental data

Supplemental data

Supplemental data

Supplemental data

Supplemental data

Supplemental data
