# Supplementary material for: The Effect of Growth Factors on Vaginal Wound Healing: A Systematic Review and Meta-analysis
Source: Tissue Eng Part B Rev. 2023 Aug 8;29(4):429–40. doi: 10.1089/ten.teb.2022.0225 (PMC10701546; doi:10.1089/ten.teb.2022.0225)
Supplement: Supplemental data [file Suppl_TableS1.pdf]

Table S1: PubMed Search (Growth Factors AND vaginal wound healing AND (in vitro OR animal studies)

(("animal experimentation"[MeSH Terms] OR "models, animal"[MeSH Terms] OR "invertebrates"[MeSH Terms] OR "Animals"[Mesh:noexp] OR "animal population groups"[MeSH Terms] OR "chordata"[MeSH Terms:noexp] OR "chordata, nonvertebrate"[MeSH Terms] OR "vertebrates"[MeSH Terms:noexp] OR "amphibians"[MeSH Terms] OR "birds"[MeSH Terms] OR "fishes"[MeSH Terms] OR "reptiles"[MeSH Terms] OR "mammals"[MeSH Terms:noexp] OR "primates"[MeSH Terms:noexp] OR "artiodactyla"[MeSH Terms] OR "carnivora"[MeSH Terms] OR "cetacea"[MeSH Terms] OR "chiroptera"[MeSH Terms] OR "elephants"[MeSH Terms] OR "hyraxes"[MeSH Terms] OR "insectivora"[MeSH Terms] OR "lagomorpha"[MeSH Terms] OR "marsupialia"[MeSH Terms] OR "monotremata"[MeSH Terms] OR "perissodactyla"[MeSH Terms] OR "rodentia"[MeSH Terms] OR "scandentia"[MeSH Terms] OR "sirenia"[MeSH Terms] OR "cingulata"[MeSH Terms] OR "haplorhini"[MeSH Terms:noexp] OR "strepsirhini"[MeSH Terms] OR "platyrrhini"[MeSH Terms] OR "tarsii"[MeSH Terms] OR "catarrhini"[MeSH Terms:noexp] OR "cercopithecidae"[MeSH Terms] OR "hylobatidae"[MeSH Terms] OR "hominidae"[MeSH Terms:noexp] OR "gorilla gorilla"[MeSH Terms] OR "pan paniscus"[MeSH Terms] OR "pan troglodytes"[MeSH Terms] OR "pongo pygmaeus"[MeSH Terms] OR ((animals[tiab] OR animal[tiab] OR mice[Tiab] OR mus[Tiab] OR mouse[Tiab] OR murine[Tiab] OR woodmouse[tiab] OR rats[Tiab] OR rat[Tiab] OR murinae[Tiab] OR muridae[Tiab] OR cottonrat[tiab] OR cottonrats[tiab] OR hamster[tiab] OR hamsters[tiab] OR cricetinae[tiab] OR rodentia[Tiab] OR rodent[Tiab] OR rodents[Tiab] OR pigs[Tiab] OR pig[Tiab] OR swine[tiab] OR swines[tiab] OR piglets[tiab] OR piglet[tiab] OR boar[tiab] OR boars[tiab] OR "sus scrofa"[tiab] OR ferrets[tiab] OR ferret[tiab] OR polecat[tiab] OR polecats[tiab] OR "mustela putorius"[tiab] OR "guinea pigs"[Tiab] OR "guinea pig"[Tiab] OR cavia[Tiab] OR callithrix[Tiab] OR marmoset[Tiab] OR marmosets[Tiab] OR cebuella[Tiab] OR hapale[Tiab] OR octodon[Tiab] OR chinchilla[Tiab] OR chinchillas[Tiab] OR gerbillinae[Tiab] OR gerbil[Tiab] OR gerbils[Tiab] OR jird[Tiab] OR jirds[Tiab] OR merione[Tiab] OR meriones[Tiab] OR rabbits[Tiab] OR rabbit[Tiab] OR hares[Tiab] OR hare[Tiab] OR diptera[Tiab] OR flies[Tiab] OR fly[Tiab] OR dipteral[Tiab] OR drosophila[Tiab] OR drosophilidae[Tiab] OR cats[Tiab] OR cat[Tiab] OR carus[Tiab] OR felis[Tiab] OR nematoda[Tiab] OR nematode[Tiab] OR nematodes[Tiab] OR sipunculida[Tiab] OR dogs[Tiab] OR dog[Tiab] OR canine[Tiab] OR canines[Tiab] OR canis[Tiab] OR sheep[Tiab] OR sheeps[Tiab] OR mouflon[Tiab] OR mouflons[Tiab] OR ovis[Tiab] OR goats[Tiab] OR goat[Tiab] OR capra[Tiab] OR capras[Tiab] OR rupicapra[Tiab] OR chamois[Tiab] OR haplorhini[Tiab] OR monkey[Tiab] OR monkeys[Tiab] OR anthropoidea[Tiab] OR anthropoids[Tiab] OR saguinus[Tiab] OR tamarin[Tiab] OR tamarins[Tiab] OR leontopithecus[Tiab] OR hominidae[Tiab] OR ape[Tiab] OR apes[Tiab] OR "pan paniscus"[Tiab] OR bonobo[Tiab] OR bonobos[Tiab] OR "pan troglodytes"[Tiab] OR gibbon[Tiab] OR gibbons[Tiab] OR siamang[Tiab] OR siamangs[Tiab] OR nomascus[Tiab] OR symphalangus[Tiab] OR chimpanzee[Tiab] OR chimpanzees[Tiab] OR prosimian[Tiab] OR prosimians[Tiab] OR "bush baby"[Tiab] OR bush babies[Tiab] OR galagos[Tiab] OR galago[Tiab] OR pongidae[Tiab] OR gorilla[Tiab] OR gorillas[Tiab] OR "pongo pygmaeus"[Tiab] OR orangutan[Tiab] OR orangutans[Tiab] OR lemur[Tiab] OR lemurs[Tiab] OR lemuridae[Tiab] OR horse[Tiab] OR horses[Tiab] OR equus[Tiab] OR cow[Tiab] OR calf[Tiab] OR bull[Tiab] OR chicken[Tiab] OR chickens[Tiab] OR gallus[Tiab] OR quail[Tiab] OR bird[Tiab] OR birds[Tiab] OR quails[Tiab] OR poultry[Tiab] OR poultries[Tiab] OR fowl[Tiab] OR fowls[Tiab] OR reptile[Tiab] OR reptilia[Tiab] OR reptiles[Tiab] OR snakes[Tiab] OR snake[Tiab] OR lizard[Tiab] OR lizards[Tiab] OR alligator[Tiab] OR alligators[Tiab] OR crocodile[Tiab] OR crocodiles[Tiab] OR turtle[Tiab] OR turtles[Tiab] OR amphibian[Tiab] OR amphibians[Tiab] OR amphibia[Tiab] OR frog[Tiab] OR frogs[Tiab] OR bombina[Tiab] OR salientia[Tiab] OR toad[Tiab] OR toads[Tiab] OR "epidalea calamita"[Tiab] OR salamander[Tiab] OR salamanders[Tiab] OR eel[Tiab] OR eels[Tiab] OR fish[Tiab] OR fishes[Tiab] OR pisces[Tiab] OR catfish[Tiab] OR catfishes[Tiab] OR siluriformes[Tiab] OR arius[Tiab] OR heteropneustes[Tiab] OR sheatfish[Tiab] OR perch[Tiab] OR perches[Tiab] OR percidae[Tiab] OR perca[Tiab] OR trout[Tiab] OR trouts[Tiab] OR

char[Tiab] OR chars[Tiab] OR salvelinus[Tiab] OR minnow[Tiab] OR cyprinidae[Tiab] OR carps[Tiab] OR carp[Tiab] OR zebrafish[Tiab] OR zebrafishes[Tiab] OR goldfish[Tiab] OR goldfishes[Tiab] OR guppy[Tiab] OR guppies[Tiab] OR chub[Tiab] OR chubs[Tiab] OR tinca[Tiab] OR barbels[Tiab] OR barbus[Tiab] OR pimephales[Tiab] OR promelas[Tiab] OR "poecilia reticulata"[Tiab] OR mullet[Tiab] OR mullets[Tiab] OR eel[Tiab] OR eels[Tiab] OR seahorse[Tiab] OR seahorses[Tiab] OR mugil curema[Tiab] OR atlantic cod[Tiab] OR shark[Tiab] OR sharks[Tiab] OR catshark[Tiab] OR anguilla[Tiab] OR salmonid[Tiab] OR salmonids[Tiab] OR whitefish[Tiab] OR whitefishes[Tiab] OR salmon[Tiab] OR salmons[Tiab] OR sole[Tiab] OR solea[Tiab] OR lamprey[Tiab] OR lampreys[Tiab] OR pumpkinseed[Tiab] OR sunfish[Tiab] OR sunfishes[Tiab] OR tilapia[Tiab] OR tilapias[Tiab] OR turbot[Tiab] OR turbots[Tiab] OR flatfish[Tiab] OR flatfishes[Tiab] OR sciuridae[Tiab] OR squirrel[Tiab] OR squirrels[Tiab] OR chipmunk[Tiab] OR chipmunks[Tiab] OR suslik[Tiab] OR susliks[Tiab] OR vole[Tiab] OR voles[Tiab] OR lemming[Tiab] OR lemmings[Tiab] OR muskrat[Tiab] OR muskrats[Tiab] OR lemmus[Tiab] OR otter[Tiab] OR otters[Tiab] OR marten[Tiab] OR martens[Tiab] OR martes[Tiab] OR weasel[Tiab] OR badger[Tiab] OR badgers[Tiab] OR ermine[Tiab] OR mink[Tiab] OR minks[Tiab] OR sable[Tiab] OR sables[Tiab] OR gulo[Tiab] OR gulos[Tiab] OR wolverine[Tiab] OR wolverines[Tiab] OR mustela[Tiab] OR llama[Tiab] OR llamas[Tiab] OR alpaca[Tiab] OR alpacas[Tiab] OR camelid[Tiab] OR camelids[Tiab] OR guanaco[Tiab] OR guanacos[Tiab] OR chiroptera[Tiab] OR chiropteras[Tiab] OR bat[Tiab] OR bats[Tiab] OR fox[Tiab] OR foxes[Tiab] OR iguana[Tiab] OR iguanas[Tiab] OR xenopus laevis[Tiab] OR parakeet[Tiab] OR parakeets[Tiab] OR parrot[Tiab] OR parrots[Tiab] OR donkey[Tiab] OR donkeys[Tiab] OR mule[Tiab] OR mules[Tiab] OR zebra[Tiab] OR zebras[Tiab] OR shrew[Tiab] OR shrews[Tiab] OR bison[Tiab] OR bisons[Tiab] OR buffalo[Tiab] OR buffaloes[Tiab] OR deer[Tiab] OR deers[Tiab] OR bear[Tiab] OR bears[Tiab] OR panda[Tiab] OR pandas[Tiab] OR "wild hog"[Tiab] OR "wild boar"[Tiab] OR fitchew[Tiab] OR fitch[Tiab] OR beaver[Tiab] OR beavers[Tiab] OR jerboa[Tiab] OR jerboas[Tiab] OR capybara[Tiab] OR capybaras[Tiab] OR canine [tiab] OR bovine [tiab] OR porcine [tiab] OR hog [tiab] OR hogs [tiab]) NOT medline[sb])) OR "Cells"[Mesh] OR "Tissues"[Mesh] OR "In Vitro Techniques"[Mesh] OR "Organoids"[Mesh] OR "Primary Cell Culture"[Mesh] OR "Spheroids, Cellular"[Mesh] OR "Diffusion Chambers, Culture"[Mesh] OR "Animal Testing Alternatives"[Mesh] OR "High-Throughput Screening Assays"[Mesh] OR "Stem Cells"[Mesh] OR "Hydrogels"[Mesh] OR "Fibroblasts"[Mesh] OR "Organ Culture Techniques"[Mesh] OR "Tissue Culture Techniques"[Mesh] OR "Tissue Engineering"[Mesh] OR "Cell Culture Techniques"[Mesh] OR "Cell- and Tissue-Based Therapy"[Mesh] OR "Tissue Culture Techniques"[Mesh] OR "animal testing alternative"[tiab] OR "animal testing alternatives"[tiab] OR "alternative to animal testing"[tiab] OR "alternatives to animal testing"[tiab] OR "animal use alternatives"[tiab] OR "animal use alternatives"[tiab] OR "animal testing reduction"[tiab] OR "in vitro"[tiab] OR "invitro"[tiab] OR "ex vivo"[tiab] OR "culture technique"[tiab] OR "culture techniques"[tiab] OR "axenic culture"[tiab] OR "axenic cultures"[tiab] OR "aseptic culture"[tiab] OR "aseptic cultures"[tiab] OR "sterile culture"[tiab] OR "sterile cultures"[tiab] OR "cell culture"[tiab] OR "cell cultures"[tiab] OR "batch culture"[tiab] OR "batch cultures"[tiab] OR "shake-flask culture"[tiab] OR "shake-flask cultures"[tiab] OR "cell engineering"[tiab] OR "cellular engineering"[tiab] OR "tissue engineering"[tiab] OR "organoid"[tiab] OR "organoids"[tiab] OR "culture diffusion chambers"[tiab] OR "semi-permeable chamber"[tiab] OR "semi-permeable chambers"[tiab] OR "semipermeable chamber"[tiab] OR "semipermeable chambers"[tiab] OR "tissue cage"[tiab] OR "tissue cages"[tiab] OR "coculture"[tiab] OR "cocultures"[tiab] OR "coculturing"[tiab] OR "cocultured"[tiab] OR "co-culture"[tiab] OR "co-cultures"[tiab] OR "co-culturing"[tiab] OR "co-cultured"[tiab] OR "cocultivation"[tiab] OR "cocultivations"[tiab] OR "co-cultivation"[tiab] OR "co-cultivations"[tiab] OR "continuous culture"[tiab] OR "continuous cultures"[tiab] OR "dissociated culture"[tiab] OR "dissociated cultures"[tiab] OR "microcarrier culture"[tiab] OR "microcarrier cultures"[tiab] OR "monolayer culture"[tiab] OR "monolayer cultures"[tiab] OR "primary culture"[tiab] OR "primary cultures"[tiab] OR "primary cell system"[tiab] OR "primary cell systems"[tiab] OR "suspension culture"[tiab] OR "suspension cultures"[tiab] OR "synchronous culture"[tiab] OR "synchronous

cultures"[tiab] OR "xeno-free culture"[tiab] OR "xeno-free cultures"[tiab] OR "erythrocyte culture"[tiab] OR "erythrocyte cultures"[tiab] OR "cultured erythrocytes"[tiab] OR "cultured erythroid cells"[tiab] OR "cultured red blood cells"[tiab] OR "RBC cultures"[tiab] OR "fibroblast culture"[tiab] OR "fibroblast cultures"[tiab] OR "yeast culture"[tiab] OR "yeast cultures"[tiab] OR "astrocyte culture"[tiab] OR "astrocyte cultures"[tiab] OR "microglial culture"[tiab] OR "microglial cultures"[tiab] OR "oligodendrocyte culture"[tiab] OR "oligodendrocyte cultures"[tiab] OR "organ culture"[tiab] OR "organ cultures"[tiab] OR "organ culturing"[tiab] OR "tissue culture"[tiab] OR "tissue cultures"[tiab] OR "tissue culturing"[tiab] OR "heart culture"[tiab] OR "heart cultures"[tiab] OR "kidney culture"[tiab] OR "kidney cultures"[tiab] OR "leukocyte culture"[tiab] OR "leukocyte cultures"[tiab] OR "cultured leucocytes"[tiab] OR "cultured leukocytes"[tiab] OR "cultured white blood cells"[tiab] OR "leucocyte culture"[tiab] OR "leucocyte cultures"[tiab] OR "lymphocyte culture"[tiab] OR "lymphocyte cultures"[tiab] OR "cultured lymphocytes"[tiab] OR "NK cultures"[tiab] OR "macrophage culture"[tiab] OR "macrophage cultures"[tiab] OR "monocyte culture"[tiab] OR "monocyte cultures"[tiab] OR "hepatocyte culture"[tiab] OR "hepatocyte cultures"[tiab] OR "liver culture"[tiab] OR "liver cultures"[tiab] OR "mixed culture"[tiab] OR "mixed cultures"[tiab] OR "muscle fiber cultures"[tiab] OR "muscle culture"[tiab] OR "muscle cultures"[tiab] OR "myocyte culture"[tiab] OR "myocyte cultures"[tiab] OR "protoplast culture"[tiab] OR "protoplast cultures"[tiab] OR "feeder cell"[tiab] OR "feeder cells"[tiab] OR "feeder layer"[tiab] OR "feeder layers"[tiab] OR "primary cell"[tiab] OR "primary cells"[tiab] OR "cultured tumor cells"[tiab] OR "cultured tumour cells"[tiab] OR "tumor culture"[tiab] OR "tumor cultures"[tiab] OR "tumour culture"[tiab] OR "tumour cultures"[tiab] OR "cancer culture"[tiab] OR "cancer cultures"[tiab] OR "tumor spheroid"[tiab] OR "tumor spheroids"[tiab] OR "tumour spheroid"[tiab] OR "tumour spheroids"[tiab] OR "embryo culture"[tiab] OR "embryo cultures"[tiab] OR "blastocyst culture"[tiab] OR "blastocyst cultures"[tiab] OR "bone marrow culture"[tiab] OR "bone marrow cultures"[tiab] OR "skin culture"[tiab] OR "skin cultures"[tiab] OR "slice culture"[tiab] OR "slice cultures"[tiab] OR "high-throughput screen"[tiab] OR "high-throughput screens"[tiab] OR "high-throughput screening"[tiab] OR "high-throughput drug screen"[tiab] OR "high-throughput drug screens"[tiab] OR "high-throughput drug screening"[tiab] OR "high-throughput biological assay"[tiab] OR "high-throughput biological assays"[tiab] OR "high-throughput chemical assay"[tiab] OR "high-throughput chemical assays"[tiab] OR vaginal fibroblast[tiab] OR "vaginal fibroblasts"[tiab] OR "vaginal cell"[tiab] OR "vaginal cells"[tiab] OR "vaginal tissue"[tiab] OR "vaginal tissues"[tiab]) AND ("Vagina"[Mesh] OR "Vaginal Diseases"[Mesh] OR vaginas[tiab] OR Vaginal Disease[tiab] OR "Vaginal Fistula"[Mesh] OR Vaginal Fistulas[tiab] OR "Suburethral Slings"[Mesh] OR Suburethral Sling[tiab] OR Transobturator Tape[tiab] OR Transobturator Tapes[tiab] OR Transobturator Suburethral Tape[tiab] OR Transobturator Suburethral Tapes[tiab] OR Trans-Obturator Tape[tiab] OR Trans Obturator Tape[tiab] OR Trans-Obturator Tapes[tiab] OR Urethral Slings[tiab] OR Urethral Sling[tiab] OR Midurethral Slings[tiab] OR Midurethral Sling[tiab] OR Mid-Urethral Slings[tiab] OR Mid-Urethral Sling[tiab] OR Vaginal Tape[tiab] OR TVT[tiab] OR TOT[tiab] OR AJUST[tiab] OR AJUSTTM[tiab] OR MiniArc[tiab] OR Mini-arc[tiab] OR Altis[tiab] OR SECUR[tiab] OR Monarc[tiab] OR Ophira[tiab] OR Solyx[tiab] OR RetroArc[tiab] OR Desara[tiab] OR Supris[tiab] OR Obtryx[tiab] OR Abbrevo[tiab] OR ARIS[tiab] OR Lynx[tiab] OR "Hysterectomy, Vaginal"[Mesh] OR Vaginal Hysterectomies[tiab] OR Vaginal Hysterectomy[tiab] OR Colpohysterectomy[tiab] OR Colpohysterectomies[tiab] OR "Episiotomy"[Mesh] OR Episiotomies[tiab] OR "Colpotomy"[Mesh] OR Colpotomies[tiab] OR Vaginotomy[tiab] OR Vaginotomies[tiab] OR "Genitalia"[Mesh] OR "Genitalia, Female"[Mesh] OR Genital Organs[tiab] OR Genital Organ[tiab] OR Genital System[tiab] OR Genital Systems[tiab] OR Genitals[tiab] OR Genital[tiab] OR Reproductive Organs[tiab] OR Reproductive Organ[tiab] OR Reproductive System[tiab] OR Reproductive Systems[tiab] OR Accessory Sex Organs[tiab] OR Accessory Sex Organ[tiab] OR Female Genitalia[tiab] OR Female Genital[tiab] OR Female Genitals[tiab] OR "Genital Neoplasms, Female"[Mesh] OR Gynecologic Neoplasms[tiab] OR Gynecologic Neoplasm[tiab] OR Female Genital Neoplasms[tiab] OR Female Genital Neoplasm[tiab] OR "Genital Diseases, Female"[Mesh] OR Gynecologic Diseases[tiab] OR Gynecologic Disease[tiab] OR Female Genital

Diseases[tiab] OR Female Genital Disease[tiab] OR "Pelvic Organ Prolapse"[Mesh] OR "Pelvic Floor Disorders"[Mesh] OR "Pelvic Floor"[Mesh] OR "Urinary Incontinence"[Mesh] OR "Urinary Incontinence, Stress"[Mesh] OR Pelvic Organ Prolapses[tiab] OR Urogenital Prolapse[tiab] OR Urogenital Prolapses[tiab] OR Vaginal Vault Prolapse[tiab] OR Vaginal Vault Prolapses[tiab] OR Pelvic Floor Disorder[tiab] OR Pelvic Floor Diseases[tiab] OR Pelvic Floor Disease[tiab] OR Pelvic Diaphragm[tiab] OR Pelvic Diaphragms[tiab] OR Urinary Stress Incontinence[tiab]) AND ("Wound Healing"[Mesh] OR Wound Healings[tiab] OR "Regeneration"[Mesh] OR Regenerations[tiab] OR Endogenous Regeneration[tiab] OR "Fibrosis"[Mesh] OR Fibroses[tiab] OR "Tissue Scaffolds"[Mesh] OR "Biocompatible Materials"[Mesh] OR "Biomimetic Materials"[Mesh] OR "Hydrogels"[Mesh] OR Tissue Scaffold[tiab] OR Tissue Scaffolding[tiab] OR Tissue Scaffoldings[tiab] OR Biocompatible Material[tiab] OR Biomaterials[tiab] OR Biomaterial[tiab] OR Bioartificial Materials[tiab] OR Bioartificial Material[tiab] OR Hemocompatible Materials[tiab] OR Hemocompatible Material[tiab] OR Biomimetic Material[tiab] OR Biomimicry Materials[tiab] OR Biomimicry Material[tiab] OR Biomimetic Device[tiab] OR Biomimicry Devices[tiab] OR Biomimicry Device[tiab] OR Biomimicry Devices[tiab] OR Hydrogel[tiab] OR In Situ Hydrogels[tiab] OR In Situ Hydrogel[tiab] OR Patterned Hydrogels[tiab] OR Patterned Hydrogel[tiab] OR "Autografts"[Mesh] OR "Bioprosthesis"[Mesh] OR "Biological Dressings"[Mesh] OR "Heterografts"[Mesh] OR "Allografts"[Mesh] OR Autograft[tiab] OR Heterograft[tiab] OR Xenografts[tiab] OR Xenograft[tiab] OR Allograft[tiab] OR Autologous Transplants[tiab] OR Autologous Transplant[tiab] OR Autotransplant[tiab] OR Bioprostheses[tiab] OR Glutaraldehyde-Stabilized Grafts[tiab] OR Glutaraldehyde Stabilized Grafts[tiab] OR Biological Dressing[tiab] OR Biologic Dressing[tiab] OR Pig Skin Dressings[tiab] OR Pig Skin Dressing[tiab] OR Amniotic Membrane Dressings[tiab] OR Amniotic Membrane Dressing[tiab] OR Allogeneic Transplants[tiab] OR Allogeneic Transplant[tiab] OR Allogeneic Grafts[tiab] OR Allogeneic Graft[tiab] OR Homografts[tiab] OR Homograft[tiab] OR Homologous Transplants[tiab] OR Homologous Transplant[tiab] OR Native Tissue Repair[tiab] OR pelvic floor repair [tiab] OR "Surgical Mesh"[Mesh] OR "Polypropylenes"[Mesh] OR "Polyglactin 910"[Mesh] OR Surgical Meshes[tiab] OR Propylene Polymers[tiab] OR Propene Polymers[tiab] OR Polypropylene[tiab] OR Prolene[tiab] OR Polypro[tiab] OR Hostalen[tiab] OR Marlex[tiab] OR Polygalactin 910[tiab] OR Vicryl[tiab] OR "Poly Lactide-Co-Glycoside"[tiab] OR Polyglactin[tiab] OR "Poly Lactide-Co-Glycolide"[tiab] OR Poly Glycolide Lactide Copolymer[tiab] OR Gynemesh[tiab] OR Polyform[tiab] OR Dexon[tiab] OR Vypro[tiab] OR Pelvicol[tiab] OR Pelvisoft[tiab] OR Pelvitex[tiab] OR Mersuture[tiab] OR Ugytex[tiab] OR UltraPro[tiab] OR SmartMesh[tiab] OR Dynamesh[tiab] or Coloplast[tiab] OR Prolift[tiab] OR SPMM[tiab] OR SPMW[tiab] OR Surgipro[tiab] OR Avaulta[tiab] OR Surgisis[tiab] OR InteXen[tiab] OR Perigee[tiab] OR Zenoderm[tiab] OR BARD[tiab] OR Matristem[tiab] OR Parietene[tiab]) AND ("intercellular signaling peptides and proteins"[MeSH] OR "Receptors, Growth Factor"[Mesh] OR Paracrine Peptide Factors[tiab] OR Paracrine Peptide Factor[tiab] OR Paracrine Protein Factors[tiab] OR Paracrine Protein Factor[tiab] OR Trophic Factor Receptors [tiab] OR Trophic Factor Receptor [tiab] OR "Epidermal Growth Factor"[MeSH] OR "EGF Family of Proteins"[MeSH] OR "ErbB Receptors"[MeSH] OR "Heparin-binding EGF-like Growth Factor"[MeSH] OR Urogastrone[tiab] OR Urogastrones[tiab] OR EGF[tiab] OR Human Urinary Gastric Inhibitor[tiab] OR beta-Urogastrone[tiab] OR Epidermal Growth Factor-Urogastrone[tiab] OR Transforming Growth Factor alpha[tiab] OR TGF-alpha[tiab] OR Epidermal Growth Factor-Like Proteins[tiab] OR Epidermal Growth Factor-Like Protein[tiab] OR Heparin-binding Epidermal Growth Factor-like Growth Factor [tiab] OR Heparin binding Epidermal Growth Factor like Growth Factor[tiab] OR Diphtheria Toxin Receptor[tiab] OR Diphtheria Toxin Receptors[tiab] OR HER Family Receptors[tiab] OR HER Family Receptor[tiab] OR ErbB-1 Receptor[tiab] OR ErbB-1 Receptors[tiab] OR c-ErbB-1 [tiab] OR ErbB 1 [tiab] OR c-erbB-1[tiab] OR c erbB 1 Protein[tiab] OR Epidermal Growth Factor Like Proteins[tiab] OR Epidermal Growth Factor Like Protein[tiab] OR "Transforming Growth Factors"[MeSH] OR "Transforming Growth Factor beta"[MeSH] OR "Transforming Growth Factor alpha"[MeSH] OR "Transforming Growth Factor beta1"[MeSH] OR "Transforming Growth Factor beta2"[MeSH] OR "Transforming Growth Factor beta3"[MeSH] OR "TGF-beta Superfamily Proteins"[MeSH] OR "Receptors,

Transforming Growth Factor beta"[MeSH] OR "Receptor, Transforming Growth Factor-beta Type II"[MeSH] OR "Receptor, Transforming Growth Factor-beta Type I"[MeSH] OR Transforming Growth Factor[tiab] OR Transforming Growth Factors[tiab] OR Milk Growth Factor[tiab] OR Milk Growth Factors[tiab] OR TGF-beta[tiab] OR TGF beta[tiab] OR TGFbeta[tiab] OR TGF-alpha[tiab] OR TGFalpha[tiab] OR TGF-beta1[tiab] OR TGF-beta-1[tiab] OR Glioblastoma Derived T Cell Suppressor Factor[tiab] OR Cartilage Inducing Factor B[tiab] OR Polyergin[tiab] OR TGF-beta2[tiab] OR TGF-beta-2[tiab] OR TGF-beta2[tiab] OR BSC 1 Cell Growth Inhibitor[tiab] OR TGF-beta-3[tiab] OR TGFB3[tiab] OR TGF-beta3[tiab] OR TGF beta3[tiab] OR TGFBR1[tiab] OR "Fibroblast Growth Factors"[MeSH] OR "Fibroblast Growth Factor 1"[MeSH] OR "Fibroblast Growth Factor 2"[MeSH] OR "Fibroblast Growth Factor 3"[MeSH] OR "Fibroblast Growth Factor 4"[MeSH] OR "Fibroblast Growth Factor 5"[MeSH] OR "Fibroblast Growth Factor 6"[MeSH] OR "Fibroblast Growth Factor 7"[MeSH] OR "Fibroblast Growth Factor 8"[MeSH] OR "Fibroblast Growth Factor 9"[MeSH] OR "Fibroblast Growth Factor 10"[MeSH] OR "Receptors, Fibroblast Growth Factor"[MeSH] OR "Receptor, Fibroblast Growth Factor, Type 1"[MeSH] OR "Receptor, Fibroblast Growth Factor, Type 2"[MeSH] OR "Receptor, Fibroblast Growth Factor, Type 3"[MeSH] OR "Receptor, Fibroblast Growth Factor, Type 4"[MeSH] OR "Receptor, Fibroblast Growth Factor, Type 5"[MeSH] OR Fibroblast Growth Factor[tiab] OR Fibroblast Growth Factors[tiab] OR FGF[tiab] OR HBGF-1[tiab] OR Fibroblast Growth Factor-1[tiab] OR Fibroblast Growth Factors-1[tiab] OR FGF-1[tiab] OR FGF1[tiab] OR Heparin-Binding Growth Factor[tiab] OR HBGF-2[tiab] OR Prostatropin[tiab] OR Prostatropins[tiab] OR FGF-2[tiab] OR FGF2[tiab] OR Fibroblast Growth Factor-2[tiab] OR Cartilage-Derived Growth Factor[tiab] OR Cartilage-Derived Growth Factors[tiab] OR Cartilage Derived Growth Factor[tiab] OR Cartilage Derived Growth Factors[tiab] OR Prostate Epithelial Cell Growth Factor[tiab] OR Hst-1 [tiab] OR kfgf[tiab] OR Keratinocyte Growth Factor[tiab] OR Keratinocyte Growth Factors[tiab] OR Palifermin[tiab] OR Androgen-Induced Growth Factor[tiab] OR Androgen Induced Growth Factor[tiab] OR Glial Activating Factor[tiab] OR Glial Activating Factors[tiab] OR Keratinocyte Growth Factor 2[tiab] OR Repifermin[tiab] OR DNA Synthesis Factor[tiab] OR DNA Synthesis Factors[tiab] OR FGFR1 [tiab] OR CD331 Antigen[tiab] OR CD331[tiab] OR FGFR2[tiab] OR CD332[tiab] OR FGFR2c[tiab] OR FGFR2b[tiab] OR FGFR3[tiab] OR CD333[tiab] OR "Vascular Endothelial Growth Factors"[MeSH] OR "Vascular Endothelial Growth Factor A"[MeSH] OR "Vascular Endothelial Growth Factor B"[MeSH] OR "Vascular Endothelial Growth Factor C"[MeSH] OR "Vascular Endothelial Growth Factor D"[MeSH] OR "Vascular Endothelial Growth Factor, Endocrine-Gland-Derived"[MeSH] OR "Receptors, Vascular Endothelial Growth Factor"[MeSH] OR "Vascular Endothelial Growth Factor Receptor-1"[MeSH] OR "Vascular Endothelial Growth Factor Receptor-2"[MeSH] OR "Vascular Endothelial Growth Factor Receptor-3"[MeSH] OR Vascular Endothelial Growth Factor[tiab] OR Vascular Endothelial Growth Factors[tiab] OR VEGFs[tiab] OR VEGF[tiab] OR Vascular Endothelial Growth Factor-A[tiab] OR Vascular Endothelial Growth Factors-A[tiab] OR VEGF-A[tiab] OR Vasculotropin[tiab] OR Vascular Permeability Factors[tiab] OR GD-VEGF[tiab] OR VEGF-B[tiab] OR Vascular Endothelial Growth Factor-B[tiab] OR VEGF-C[tiab] OR Vascular Endothelial Growth Factor-C[tiab] OR Vascular Endothelial Growth Factors-C[tiab] OR VEGF-D[tiab] OR Vascular Endothelial Growth Factor-D[tiab] OR c-fos-Induced Growth Factor[tiab] OR FIGF[tiab] OR Prokineticin 1[tiab] OR EG-VEGF[tiab] OR VPF Receptor[tiab] OR VPF Receptors[tiab] OR Endothelial Growth Factor Receptor[tiab] OR Endothelial Growth Factor Receptors[tiab] OR FLT[tiab] OR FLT1[tiab] OR FLT-1[tiab] OR VEGFR-1[tiab] OR fms-Like Tyrosine Kinase[tiab] OR KDR Tyrosine Kinase[tiab] OR KDR Tyrosine Kinases[tiab] OR VEGFR-2[tiab] OR Fetal Liver Kinase-1[tiab] OR Fetal Liver Kinase 1[tiab] OR Kinase Insert Domain Receptor[tiab] OR Flk-1[tiab] OR Flk 1[tiab] OR Flt-4[tiab] OR Flt 4[tiab] OR VEGFR-3[tiab] OR "Nerve Growth Factor"[MeSH] OR "Receptor, Nerve Growth Factor"[MeSH] OR "Receptors, Nerve Growth Factor"[MeSH] OR "Nuclear Receptor Subfamily 4, Group A, Member 1"[MeSH] OR "Glial Cell Line-Derived Neurotrophic Factor"[MeSH] OR NGF[tiab] OR Nerve Growth Factor[tiab] OR Nerve Growth Factors[tiab] OR Neurotropin Receptor[tiab] OR Neurotropin Receptors[tiab] OR Neurotrophin Receptor[tiab] OR Neurotrophin Receptors[tiab] OR NGFR[tiab] OR GP80 LNGFR[tiab]

OR Neurotrophic Factor[tiab] OR Orphan Nuclear Receptor[tiab] OR NR4A1[tiab] OR NAK1[tiab] OR GDNF[tiab] OR "Platelet-Derived Growth Factor"[MeSH] OR "Becaplermin"[MeSH] OR "Receptor, Platelet-Derived Growth Factor beta"[MeSH] OR "Receptor, Platelet-Derived Growth Factor alpha"[MeSH] OR "Receptors, Platelet-Derived Growth Factor"[MeSH] OR Platelet Derived Growth Factor[tiab] OR Platelet Derived Growth Factors[tiab] OR Platelet-Derived Growth Factors[tiab] OR PDGF[tiab] OR rPDGF-BB[tiab] OR PDGF-BB[tiab] OR rhPDGF-BB[tiab] OR Regranex[tiab] OR CD140b[tiab] OR PDGFRB[tiab] OR PDGFR1[tiab] OR PDGFR[tiab] OR PDGFRalpha[tiab] OR PDGF-R-alpha[tiab] OR CD140a[tiab] OR PDGFR2[tiab] OR "Somatomedins"[Mesh] OR "Insulin-Like Growth Factor I"[MeSH] OR "Insulin-Like Growth Factor Binding Proteins"[MeSH] OR "Insulin-Like Growth Factor Binding Protein 1"[MeSH] OR "Insulin-Like Growth Factor Binding Protein 2"[MeSH] OR "Insulin-Like Growth Factor Binding Protein 3"[MeSH] OR "Insulin-Like Growth Factor Binding Protein 4"[MeSH] OR "Insulin-Like Growth Factor Binding Protein 5"[MeSH] OR "Insulin-Like Growth Factor Binding Protein 6"[MeSH] OR "Insulin-Like Growth Factor II"[MeSH] OR "Receptor, IGF Type 2"[MeSH] OR "Receptors, Somatomedin"[MeSH] OR "Receptor, IGF Type 1"[MeSH] OR "Pregnancy-Associated Plasma Protein-A"[MeSH] OR Sulfation Factor[tiab] OR Sulfation Factors[tiab] OR Insulin-Like Growth Factors[tiab] OR Insulin-Like Growth Factor[tiab] OR Somatomedin[tiab] OR Somatomedins[tiab] OR Insulin Like Growth Factor[tiab] OR Insulin Like Growth Factors[tiab] OR Insulin-Like-Growth-Factor[tiab] OR Insulin-Like-Growth-Factors[tiab] OR IGF[tiab] OR IGF-I-SmC[tiab] OR IGF-1[tiab] OR IGF-I[tiab] OR Somatomedin-Binding Proteins[tiab] OR Somatomedin-Binding Protein[tiab] OR IGFBP[tiab] OR IGF-Binding Proteins[tiab] OR IGF-Binding Protein[tiab] OR IGFBP-1[tiab] OR IGFBP-2[tiab] OR IGFBP-3[tiab] OR IGFBP-4[tiab] OR IGFBP-5[tiab] OR IGFBP-6[tiab] OR IGF-II[tiab] OR Multiplication-Stimulating Factor[tiab] OR Multiplication Stimulating Factor[tiab] OR IGF-2[tiab] OR Mannose-6-Phosphate Receptor[tiab] OR Mannose-6-Phosphate Receptors[tiab] OR Mannose 6 Phosphate Receptor[tiab] OR Mannose 6 Phosphate Receptors[tiab] OR Pregnancy Associated Plasma Protein A[tiab] OR "Connective Tissue Growth Factor"[MeSH] OR "Hepatocyte Growth Factor"[MeSH] OR "Placenta Growth Factor"[MeSH] OR "Endothelial Growth Factors"[MeSH] OR "Amphiregulin"[MeSH] OR "Stem Cell Factor"[MeSH] OR "Hematopoietic Cell Growth Factors"[MeSH] OR "Receptors, Colony-Stimulating Factor"[MeSH] OR "Bone Morphogenetic Proteins"[MeSH] OR IGF-Binding Protein-Related Protein-2[tiab] OR IGF-Binding Protein-Related Proteins-2[tiab] OR IGFBP-rP2[tiab] OR CCN2[tiab] OR IGFBP-8[tiab] OR Hepatocyte Growth Factors[tiab] OR HGF[tiab] OR Scatter Factor[tiab] OR Scatter Factors[tiab] OR Hepatopoietin[tiab] OR Hepatopoietins[tiab] OR Placenta Growth Factors[tiab] OR PLGF[tiab] OR PLGF-1[tiab] OR PLGF-2[tiab] OR PLGF-3[tiab] OR PLGF-4[tiab] OR Endo-GF[tiab] OR Endothelial Growth Factor[tiab] OR ECDGF[tiab] OR beta-Endothelial Growth Factor[tiab] OR alpha-Endothelial Growth Factor[tiab] OR alpha-Endothelial Growth Factors[tiab] OR Schwannoma-derived Growth Factor[tiab] OR Schwannoma derived Growth Factor[tiab] OR Steel Factor[tiab] OR Mast Cell Growth Factor[tiab] OR Mast Cell Growth Factors[tiab] OR c-kit[tiab] OR c kit[tiab] OR CGF[tiab] OR Hematopoietins[tiab] OR Hematopoietin[tiab] OR Hematopoietic Growth Factor[tiab] OR Hematopoietic Growth Factors[tiab] OR Hematopoietic Stem Cell-Activating Factors[tiab] OR Colony-Stimulating Factor[tiab] OR Colony Stimulating Factor[tiab] OR CSF OR Bone Morphogenetic Protein[tiab] OR Bone Morphogenetic Proteins[tiab])
