# Supplementary material for: The Effect of Growth Factors on Vaginal Wound Healing: A Systematic Review and Meta-analysis
Source: Tissue Eng Part B Rev. 2023 Aug 8;29(4):429–40. doi: 10.1089/ten.teb.2022.0225 (PMC10701546; doi:10.1089/ten.teb.2022.0225)
Supplement: Supplemental data [file Suppl_TableS2.pdf]

**Table S2: EMBASE search string animal/in vitro studies AND vaginal wound healing AND Growth factors**

(exp animal experiment/ or exp animal model/ or exp experimental animal/ or exp transgenic animal/ or exp male animal/ or exp female animal/ or exp juvenile animal/ OR animal/ OR chordata/ OR vertebrate/ OR tetrapod/ OR exp fish/ OR amniote/ OR exp amphibia/ OR mammal/ OR exp reptile/ OR exp sauropsid/ OR therian/ OR exp monotremate/ OR placental mammals/ OR exp marsupial/ OR Euarchontoglires/ OR exp Afrotheria/ OR exp Boreoeutheria/ OR exp Laurasiatheria/ OR exp Xenarthra/ OR primate/ OR exp Dermoptera/ OR exp Glires/ OR exp Scandentia/ OR Haplorhini/ OR exp prosimian/ OR simian/ OR exp tarsiiform/ OR Catarrhini/ OR exp Platyrrhini/ OR ape/ OR exp Cercopithecidae/ OR hominid/ OR exp hylobatidae/ OR exp chimpanzee/ OR exp gorilla/ OR exp orang utan/ OR (animal OR animals OR pisces OR fish OR fishes OR catfish OR catfishes OR sheatfish OR silurus OR arius OR heteropneustes OR clarias OR gariepinus OR fathead minnow OR fathead minnows OR pimephales OR promelas OR cichlidae OR trout OR trouts OR char OR chars OR salvelinus OR salmo OR oncorhynchus OR guppy OR guppies OR millionfish OR poecilia OR goldfish OR goldfishes OR carassius OR auratus OR mullet OR mullets OR mugil OR curema OR shark OR sharks OR cod OR cods OR gadus OR morhua OR carp OR carps OR cyprinus OR carpio OR killifish OR eel OR eels OR anguilla OR zander OR sander OR lucioperca OR stizostedion OR turbot OR turbots OR psetta OR flatfish OR flatfishes OR plaice OR pleuronectes OR platessa OR tilapia OR tilapias OR oreochromis OR sarotherodon OR common sole OR dover sole OR solea OR zebrafish OR zebrafishes OR danio OR rerio OR seabass OR dicentrarchus OR labrax OR morone OR lamprey OR lampreys OR petromyzon OR pumpkinseed OR pumpkinseeds OR lepomis OR gibbosus OR herring OR clupea OR harengus OR amphibia OR amphibian OR amphibians OR anura OR salientia OR frog OR frogs OR rana OR toad OR toads OR bufo OR xenopus OR laevis OR bombina OR epidalea OR calamita OR salamander OR salamanders OR newt OR newts OR triturus OR reptilia OR reptile OR reptiles OR bearded dragon OR pogona OR vitticeps OR iguana OR iguanas OR lizard OR lizards OR anguis fragilis OR turtle OR turtles OR snakes OR snake OR aves OR bird OR birds OR quail OR quails OR coturnix OR bobwhite OR colinus OR virginianus OR poultry OR poultries OR fowl OR fowls OR chicken OR chickens OR gallus OR zebra finch OR taeniopygia OR guttata OR canary OR canaries OR serinus OR canaria OR parakeet OR parakeets OR grasskeet OR parrot OR parrots OR psittacine OR psittacines OR shelduck OR tadorna OR goose OR geese OR branta OR leucopsis OR woodlark OR lullula OR flycatcher OR ficedula OR hypoleuca OR dove OR doves OR geopelia OR cuneata OR duck OR ducks OR greylag OR graylag OR anser OR harrier OR circus pygargus OR red knot OR great knot OR calidris OR canutus OR godwit OR limosa OR lapponica OR meleagris OR gallopavo OR jackdaw OR corvus OR monedula OR ruff OR philomachus OR pugnax OR lapwing OR peewit OR plover OR vanellus OR swan OR cygnus OR columbianus OR bewickii OR gull OR chroicocephalus OR ridibundus OR albifrons OR great tit OR parus OR aythya OR fuligula OR streptopelia OR risoria OR spoonbill OR platalea OR leucorodia OR blackbird OR turdus OR merula OR blue tit OR cyanistes OR pigeon OR pigeons OR columba OR pintail OR anas OR starling OR sturnus OR owl OR athene noctua OR pochard OR ferina OR cockatiel OR nymphicus OR hollandicus OR skylark OR alauda OR tern OR sterna OR teal OR crecca OR oystercatcher OR haematopus OR ostralegus OR shrew OR shrews OR sorex OR araneus OR crocidura OR russula OR european mole OR talpa OR chiroptera OR bat OR bats OR eptesicus OR serotinus OR myotis OR dasycneme OR daubentonii OR pipistrelle OR pipistrellus OR cat OR cats OR felis OR catus OR feline OR dog OR dogs OR canis OR canine OR canines OR otter OR otters OR lutra OR badger OR badgers OR meles OR fitchew OR fitch OR foumart or foulmart OR ferrets OR ferret OR polecat OR polecats OR mustela OR putorius OR weasel OR weasels OR fox OR foxes OR vulpes OR common seal OR phoca OR vitulina OR grey seal OR halichoerus OR horse OR horses OR equus OR equine OR equidae OR donkey OR donkeys OR mule OR mules OR pig OR pigs OR swine OR swines OR hog OR hogs OR boar OR boars OR porcine OR piglet OR piglets OR sus OR scrofa OR llama OR llamas OR lama OR glama OR deer OR deers OR cervus OR elaphus OR cow OR cows OR bos taurus OR bos indicus OR bovine OR bull OR bulls OR cattle OR bison OR bisons OR sheep OR sheeps OR ovis aries OR ovine OR lamb OR lambs OR mouflon OR mouflons OR goat OR goats OR

capra OR caprine OR chamois OR rupicapra OR leporidae OR lagomorpha OR lagomorph OR rabbit OR rabbits OR oryctolagus OR cuniculus OR laprine OR hares OR lepus OR rodentia OR rodent OR rodents OR murinae OR mouse OR mice OR mus OR musculus OR murine OR woodmouse OR apodemus OR rat OR rats OR rattus OR norvegicus OR guinea pig OR guinea pigs OR cavia OR porcellus OR hamster OR hamsters OR mesocricetus OR cricetulus OR cricetus OR gerbil OR gerbils OR jird OR jirds OR meriones OR unguiculatus OR jerboa OR jerboas OR jaculus OR chinchilla OR chinchillas OR beaver OR beavers OR castor fiber OR castor canadensis OR sciuridae OR squirrel OR squirrels OR sciurus OR chipmunk OR chipmunks OR marmot OR marmots OR marmota OR suslik OR susliks OR spermophilus OR cynomys OR cottonrat OR cottonrats OR sigmodon OR vole OR voles OR microtus OR myodes OR glareolus OR primate OR primates OR prosimian OR prosimians OR lemur OR lemurs OR lemuridae OR loris OR bush baby OR bush babies OR bushbaby OR bushbabies OR galago OR galagos OR anthropoidea OR anthropoids OR simian OR simians OR monkey OR monkeys OR marmoset OR marmosets OR callithrix OR cebuella OR tamarin OR tamarins OR saguinus OR leontopithecus OR squirrel monkey OR squirrel monkeys OR saimiri OR night monkey OR night monkeys OR owl monkey OR owl monkeys OR douroucoulis OR aotus OR spider monkey OR spider monkeys OR ateles OR baboon OR baboons OR papio OR rhesus monkey OR macaque OR macaca OR mulatta OR cynomolgus OR fascicularis OR green monkey OR green monkeys OR chlorocebus OR vervet OR vervets OR pygerythrus OR hominoidea OR ape OR apes OR hylobatidae OR gibbon OR gibbons OR siamang OR siamangs OR nomascus OR symphalangus OR hominidae OR orangutan OR orangutans OR pongo OR chimpanzee OR chimpanzees OR pan troglodytes OR bonobo OR bonobos OR pan paniscus OR gorilla OR gorillas OR troglodytes).ti,ab. OR Exp cells/ OR exp tissues/ OR exp in vitro study/ OR exp organoid/ OR exp cell culture/ OR primary cell culture/ OR tumor spheroid/ OR diffusion chamber/ OR exp animal testing alternative/ OR high throughput screening/ OR exp stem cell/ OR exp fibroblast/ OR exp fibroblast cell line/ OR organ culture technique/ OR tissue culture/ OR tissue engineering/ OR exp cell culture technique/ OR exp cell therapy/ OR ("animal testing alternative" OR "animal testing alternatives" OR "alternative to animal testing" OR "alternatives to animal testing" OR "animal use alternatives" OR "animal use alternatives" OR "animal testing reduction" OR "in vitro" OR "invitro" OR "ex vivo" OR "culture technique" OR "culture techniques" OR "axenic culture" OR "axenic cultures" OR "aseptic culture" OR "aseptic cultures" OR "sterile culture" OR "sterile cultures" OR "cell culture" OR "cell cultures" OR "batch culture" OR "batch cultures" OR "shake-flask culture" OR "shake-flask cultures" OR "cell engineering" OR "cellular engineering" OR "tissue engineering" OR "organoid" OR "organoids" OR "culture diffusion chambers" OR "semi-permeable chamber" OR "semi-permeable chambers" OR "semipermeable chamber" OR "semipermeable chambers" OR "tissue cage" OR "tissue cages" OR "coculture" OR "cocultures" OR "coculturing" OR "cocultured" OR "co-culture" OR "co-cultures" OR "co-culturing" OR "co-cultured" OR "cocultivation" OR "cocultivations" OR "co-cultivation" OR "co-cultivations" OR "continuous culture" OR "continuous cultures" OR "dissociated culture" OR "dissociated cultures" OR "microcarrier culture" OR "microcarrier cultures" OR "monolayer culture" OR "monolayer cultures" OR "primary culture" OR "primary cultures" OR "primary cell system" OR "primary cell systems" OR "suspension culture" OR "suspension cultures" OR "synchronous culture" OR "synchronous cultures" OR "xeno-free culture" OR "xeno-free cultures" OR "erythrocyte culture" OR "erythrocyte cultures" OR "cultured erythrocytes" OR "cultured erythroid cells" OR "cultured red blood cells" OR "RBC cultures" OR "fibroblast culture" OR "fibroblast cultures" OR "yeast culture" OR "yeast cultures" OR "astrocyte culture" OR "astrocyte cultures" OR "microglial culture" OR "microglial cultures" OR "oligodendrocyte culture" OR "oligodendrocyte cultures" OR "organ culture" OR "organ cultures" OR "organ culturing" OR "tissue culture" OR "tissue cultures" OR "tissue culturing" OR "heart culture" OR "heart cultures" OR "kidney culture" OR "kidney cultures" OR "leukocyte culture" OR "leukocyte cultures" OR "cultured leucocytes" OR "cultured leukocytes" OR "cultured white blood cells" OR "leucocyte culture" OR "leucocyte cultures" OR "lymphocyte culture" OR "lymphocyte cultures" OR "cultured lymphocytes" OR "NK cultures" OR "macrophage culture" OR "macrophage cultures" OR "monocyte culture" OR "monocyte cultures" OR "hepatocyte culture" OR "hepatocyte cultures" OR "liver culture" OR "liver cultures" OR "mixed culture" OR "mixed cultures"

OR "muscle fiber cultures" OR "muscle culture" OR "muscle cultures" OR "myocyte culture" OR "myocyte cultures" OR "protoplast culture" OR "protoplast cultures" OR "feeder cell" OR "feeder cells" OR "feeder layer" OR "feeder layers" OR "primary cell" OR "primary cells" OR "cultured tumor cells" OR "cultured tumour cells" OR "tumor culture" OR "tumor cultures" OR "tumour culture" OR "tumour cultures" OR "cancer culture" OR "cancer cultures" OR "tumor spheroid" OR "tumor spheroids" OR "tumour spheroid" OR "tumour spheroids" OR "embryo culture" OR "embryo cultures" OR "blastocyst culture" OR "blastocyst cultures" OR "bone marrow culture" OR "bone marrow cultures" OR "skin culture" OR "skin cultures" OR "slice culture" OR "slice cultures" OR "high-throughput screen" OR "high-throughput screens" OR "high-throughput screening" OR "high-throughput drug screen" OR "high-throughput drug screens" OR "high-throughput drug screening" OR "high-throughput biological assay" OR "high-throughput biological assays" OR "high-throughput chemical assay" OR "high-throughput chemical assays" OR vaginal fibroblast OR "vaginal fibroblasts" OR "vaginal cell" OR "vaginal cells" OR "vaginal tissue" OR "vaginal tissues" ).ti,ab.) AND ((Exp vagina/ OR exp vagina disease/ OR exp uterus disease/ OR exp vulva disease/ OR exp vulvovaginal disease/ OR vagina reconstruction/ OR exp vagina tissue/ OR (vagina OR vaginas OR gynecologic disease OR gynaecologic disease OR gynecological disease OR gynaecological disease OR gynecologic diseases OR female genital disease OR female genital diseases OR vaginal fistula OR vaginal fistulas).ti,ab. OR exp suburethral sling/ OR (Suburethral Sling OR Suburethral Slings OR Transobturator Tape OR Transobturator Tapes OR Transobturator Suburethral Tape OR Transobturator Suburethral Tapes OR Trans-Obturator Tape OR Trans Obturator Tape OR Trans-Obturator Tapes OR Urethral Slings OR Urethral Sling OR Midurethral Slings OR Midurethral Sling OR Mid-Urethral Slings OR Mid-Urethral Sling OR Vaginal Tape OR TVT OR TOT OR AJUST OR AJUSTTM OR MiniArc OR Mini-arc OR Altis OR SECUR OR Monarc OR Ophira OR Solyx OR RetroArc OR Desara OR Supris OR Obtryx OR Abbrevo OR ARIS OR Lynx ).ti,ab. OR vaginal hysterectomy/ OR episiotomy/ OR colpotomy/ OR (Vaginal Hysterectomies OR Vaginal Hysterectomy OR Colpohysterectomy OR Colpohysterectomies OR Episiotomies OR Colpotomies OR Vaginotomy OR Vaginotomies).ti,ab. OR pelvis floor/ OR exp pelvic organ prolapse/ OR exp pelvic floor disorder/ OR exp urine incontinence/ OR (Pelvic Organ Prolapse OR Pelvic Organ Prolapses OR POP OR Urogenital Prolapse OR Urogenital Prolapses OR Vaginal Vault Prolapse OR Vaginal Vault Prolapses OR Pelvic Floor Disorder OR Pelvic Floor Diseases OR Pelvic Floor Disease OR Pelvic Diaphragm OR Pelvic Diaphragms OR Stress urinary incontinence OR Urinary Stress Incontinence).ti,ab.) AND (exp wound healing/ OR exp regeneration/ OR exp tissue engineering/ OR (Wound Healing OR Regeneration OR Wound Healings OR Regenerations).ti,ab. OR tissue scaffold/ OR exp biomaterial/ OR biomimetic material/ OR exp hydrogel/ OR exp wound dressing/ OR (Tissue Scaffold OR Tissue Scaffolds OR Tissue Scaffolding OR Tissue Scaffoldings OR Biocompatible Material OR Biomaterial OR Biomaterials OR Bioartificial Materials OR Bioartificial Material OR Hemocompatible Materials OR Hemocompatible Material OR Biomimetic Material OR Biomimicry Materials OR Biomimicry Material OR Biomimetic Device OR biomimetic devices OR Biomimicry Devices OR Biomimicry Device OR Hydrogel OR Hydrogels).ti,ab. OR exp autograft/ OR exp bioprosthesis/ OR exp xenograft/ OR exp allograft/ OR (Autograft OR Autografts OR Heterograft OR Heterografts OR Xenografts OR Xenograft OR Allograft OR Allografts OR Autologous Transplants OR Autologous Transplant OR Autotransplant OR Bioprostheses OR Glutaraldehyde-Stabilized Grafts OR Glutaraldehyde Stabilized Grafts OR Biological Dressing OR Biologic Dressing OR Pig Skin Dressings OR Pig Skin Dressing OR Amniotic Membrane Dressings OR Amniotic Membrane Dressing OR Allogeneic Transplants OR Allogeneic Transplant OR Allogeneic Grafts OR Allogeneic Graft OR Homografts OR Homograft OR Homologous Transplants OR Homologous Transplant OR Native Tissue Repair).ti,ab. OR exp surgical mesh/ OR polypropylene/ OR polyglactin/ OR (Surgical Meshes OR Propylene Polymers OR Propene Polymers OR Polypropylene OR Prolene OR Polypro OR Hostalen OR Marlex OR Polygalactin 910 OR Vicryl OR "Poly Lactide-Co-Glycoside" OR Polyglactin OR "Poly Lactide-Co-Glycolide" OR Poly Glycolide Lactide Copolymer OR Gynemesh OR Polyform OR Dexon OR Vypro OR Pelvicol OR Pelvisoft OR Pelvitex OR Mersuture OR Ugytex OR UltraPro OR SmartMesh OR Dynamesh or Coloplast OR Prolift OR SPMM OR SPMW OR Surgipro OR Avaulta

OR Surgisis OR InteXen OR Perigee OR Zenoderm OR BARD OR Matristem OR Parietene).ti,ab.)) AND (Exp growth factor/ OR exp growth factor receptor/ OR (Growth Factor OR Growth Factors OR Paracrine Peptide Factors OR Paracrine Peptide Factor OR Paracrine Protein Factors OR Paracrine Protein Factor OR Trophic Factor Receptors OR Trophic Factor Receptor).ti,ab. OR (Urogastrone OR Urogastrones OR EGF OR Human Urinary Gastric Inhibitor OR beta-Urogastrone OR Epidermal Growth Factor-Urogastrone OR TGF-alpha OR Epidermal Growth Factor-Like Proteins OR Epidermal Growth Factor-Like Protein OR Heparin-binding Epidermal Growth Factor-like Growth Factor OR Diphtheria Toxin Receptor OR Diphtheria Toxin Receptors OR HER Family Receptors OR HER Family Receptor OR ErbB-1 Receptor OR ErbB-1 Receptors OR c-ErbB-1 OR ErbB 1 OR c-erbB-1 OR c erbB 1 Protein).ti,ab. OR transforming growth factor beta receptor 1/ OR transforming growth factor beta receptor 2/ OR transforming growth factor beta receptor 3/ OR (TGF-beta OR TGF beta OR TGFbeta OR TGF-alpha OR TGFalpha OR TGF-beta1 OR TGF-beta-1 OR Glioblastoma Derived T Cell Suppressor Factor OR Cartilage Inducing Factor B OR Polyergin OR TGF-beta2 OR TGF-beta-2 OR TGF-beta2 OR BSC 1 Cell Growth Inhibitor OR TGF-beta-3 OR TGFB3 OR TGF-beta3 OR TGF beta3 OR TGFBR1).ti,ab. OR (FGF OR HBGF-1 OR Fibroblast Growth Factor-1 OR Fibroblast Growth Factors-1 OR FGF-1 OR FGF1 OR HBGF-2 OR Prostatropin OR Prostatropins OR FGF-2 OR FGF2 OR Fibroblast Growth Factor-2 OR Hst-1 OR kfgf OR Palifermin OR Glial Activating Factor OR Glial Activating Factors OR Keratinocyte Growth Factor 2 OR Repifermin OR DNA Synthesis Factor OR DNA Synthesis Factors OR FGFR1 OR CD331 Antigen OR CD331 OR FGFR2 OR CD332 OR FGFR2c OR FGFR2b OR FGFR3 OR CD333).ti,ab. OR endocrine gland derived vascular endothelial growth factor/ OR (VEGFs OR VEGF OR VEGF-A OR Vasculotropin OR Vascular Permeability Factors OR GD-VEGF OR VEGF-B OR Vascular Endothelial Growth Factor-B OR VEGF-C OR Vascular Endothelial Growth Factor-C OR Vascular Endothelial Growth Factors-C OR VEGF-D OR Vascular Endothelial Growth Factor-D OR c-fos-Induced Growth Factor OR FIGF OR Prokineticin 1 OR EG-VEGF OR VPF Receptor OR VPF Receptors OR FLT OR FLT1 OR FLT-1 OR VEGFR-1 OR fms-Like Tyrosine Kinase OR KDR Tyrosine Kinase OR KDR Tyrosine Kinases OR VEGFR-2 OR Fetal Liver Kinase-1 OR Fetal Liver Kinase 1 OR Kinase Insert Domain Receptor OR Flk-1 OR Flk 1 OR Flt-4 OR Flt 4 OR VEGFR-3).ti,ab. OR nerve growth factor/ OR nerve growth factor receptor/ OR brain derived neurotrophic factor/ OR nerve growth factor beta subunit/ OR (NGF OR Neurotropin Receptor OR Neurotropin Receptors OR Neurotrophin Receptor OR Neurotrophin Receptors OR NGFR OR GP80 LNGFR OR Neurotrophic Factor OR Orphan Nuclear Receptor OR NR4A1 OR NAK1 OR GDNF).ti,ab. OR becaplermin/ OR (PDGF OR rPDGF-BB OR PDGF-BB OR rhPDGF-BB OR Regranex OR CD140b OR PDGFRB OR PDGFR1 OR PDGFR OR PDGFRalpha OR PDGF-R-alpha OR CD140a OR PDGFR2).ti,ab. OR exp somatomedin/ OR exp somatomedin binding protein/ OR (Sulfation Factor OR Sulfation Factors OR Somatomedin OR Somatomedins OR Insulin-Like-Growth-Factor OR Insulin-Like-Growth-Factors OR IGF OR IGF-I-SmC OR IGF-1 OR IGF-I OR Somatomedin-Binding Proteins OR Somatomedin-Binding Protein OR IGFBP OR IGF-Binding Proteins OR IGF-Binding Protein OR IGFBP-1 OR IGFBP-2 OR IGFBP-3 OR IGFBP-4 OR IGFBP-5 OR IGFBP-6 OR IGF-II OR Multiplication-Stimulating Factor OR Multiplication Stimulating Factor OR IGF-2 OR Mannose-6-Phosphate Receptor OR Mannose-6-Phosphate Receptors OR Mannose 6 Phosphate Receptor OR Mannose 6 Phosphate Receptors OR Pregnancy Associated Plasma Protein A).ti,ab. OR stem cell factor/ OR colony stimulating factor/ OR (IGF-Binding Protein-Related Protein-2 OR IGF-Binding Protein-Related Proteins-2 OR IGFBP-rP2 OR CCN2 OR IGFBP-8 OR Scatter Factor OR Scatter Factors OR Hepatopoietin OR Hepatopoietins OR PLGF OR PLGF-1 OR PLGF-2 OR PLGF-3 OR PLGF-4 OR Endo-GF OR ECDGF OR Steel Factor OR c-kit OR c kit OR CGF OR Hematopoietins OR Hematopoietin OR Hematopoietic Stem Cell-Activating Factors OR Colony-Stimulating Factor OR Colony Stimulating Factor OR CSF OR Bone Morphogenetic Protein OR Bone Morphogenetic Proteins).ti,ab.)
