# Supplementary material for: The Effect of Growth Factors on Vaginal Wound Healing: A Systematic Review and Meta-analysis
Source: Tissue Eng Part B Rev. 2023 Aug 8;29(4):429–40. doi: 10.1089/ten.teb.2022.0225 (PMC10701546; doi:10.1089/ten.teb.2022.0225)
Supplement: Supplemental data [file Suppl_TableS3.pdf]

Table S3: Adapted OHAT tool questions

A	"Was the administered dose adequately randomized?"
B	"Was allocation to study groups adequately concealed?"
C	"Were experimental conditions identical across study groups?"
D	"Were research personnel blinded to the study group during the study?"
E	"Were outcome data complete without attrition or exclusion from analysis?"
F	"Can we be confident in the treatment characterization?"
G	"Can we be confident in the outcome assessment (including blinding of outcome assessors)?"
H	"Were all measured outcomes reported?"
I	"Were there no other potential threats to internal validity?"
J	"Were there no other quality threats that could interfere with the validity of the study?"
