# Supplementary material for: The Effect of Growth Factors on Vaginal Wound Healing: A Systematic Review and Meta-analysis
Source: Tissue Eng Part B Rev. 2023 Aug 8;29(4):429–40. doi: 10.1089/ten.teb.2022.0225 (PMC10701546; doi:10.1089/ten.teb.2022.0225)
Supplement: Supplemental data [file Suppl_FigS1.pdf]

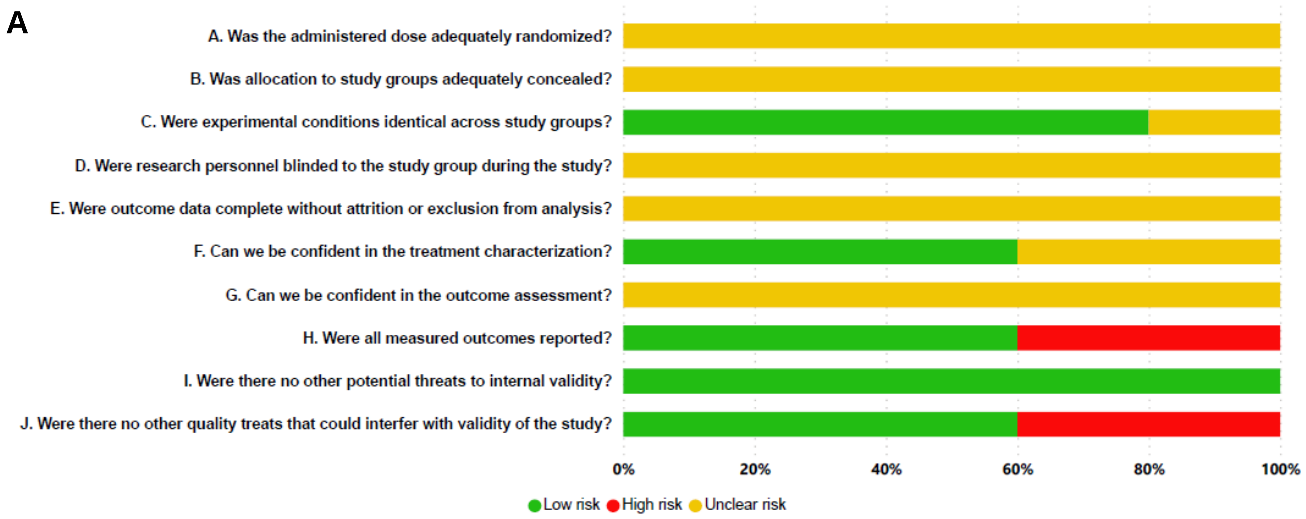

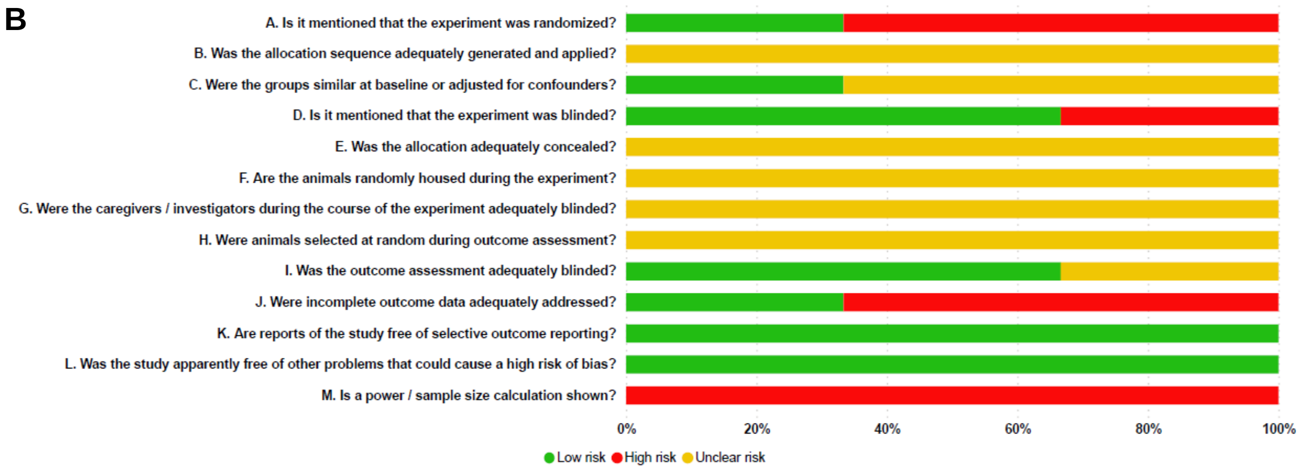

Figure S1: Quality assessment of the *in vitro* (A) and *in vivo* studies (B). The quality of the studies is assessed based on a high risk (red), low risk (green) or unclear risk (yellow) of bias. (B) Question A and D are based on reporting, whereby green indicates ‘reported’ and red ‘unreported’.
