# Supplementary figures and images for: The Effect of Growth Factors on Vaginal Wound Healing: A Systematic Review and Meta-analysis
Source: Tissue Eng Part B Rev. 2023 Aug 8;29(4):429–40. doi: 10.1089/ten.teb.2022.0225 (PMC10701546; doi:10.1089/ten.teb.2022.0225)

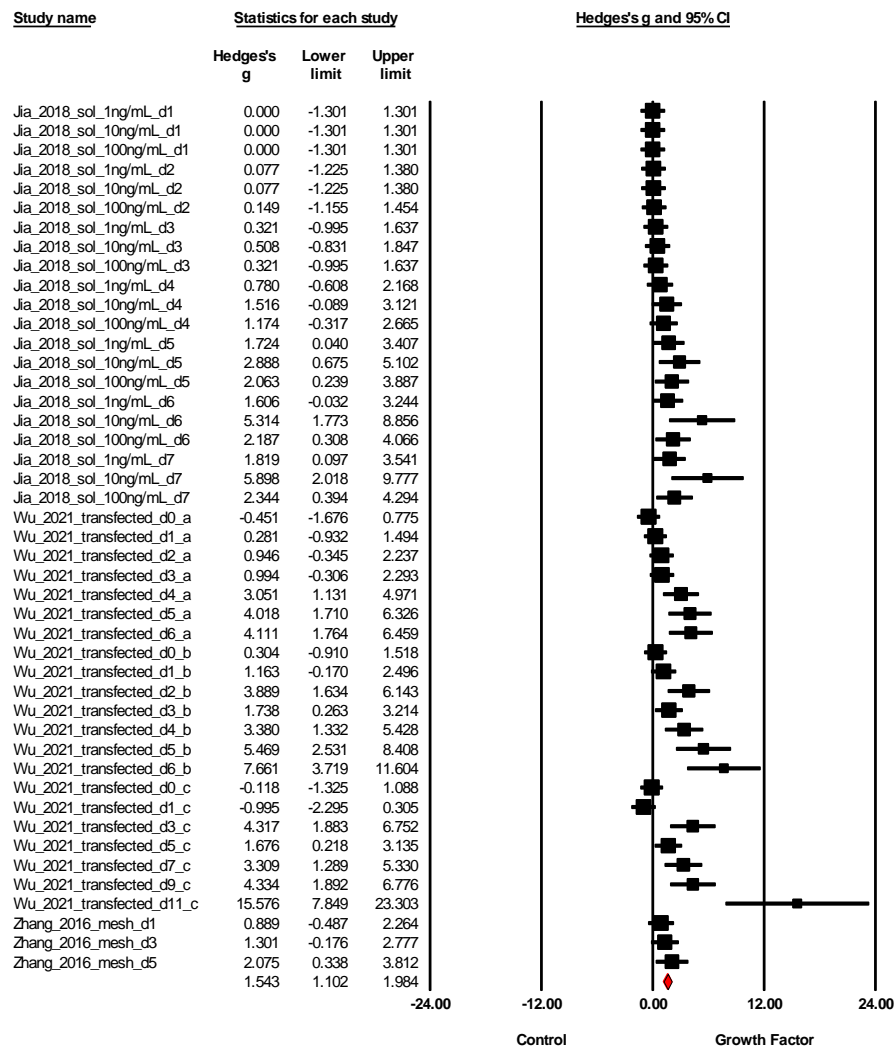

Figure S2: Forest plot displaying the effect of bFGF on proliferation *in vitro*.

Supplement: Supplemental data [file Suppl_FigS2.pdf]

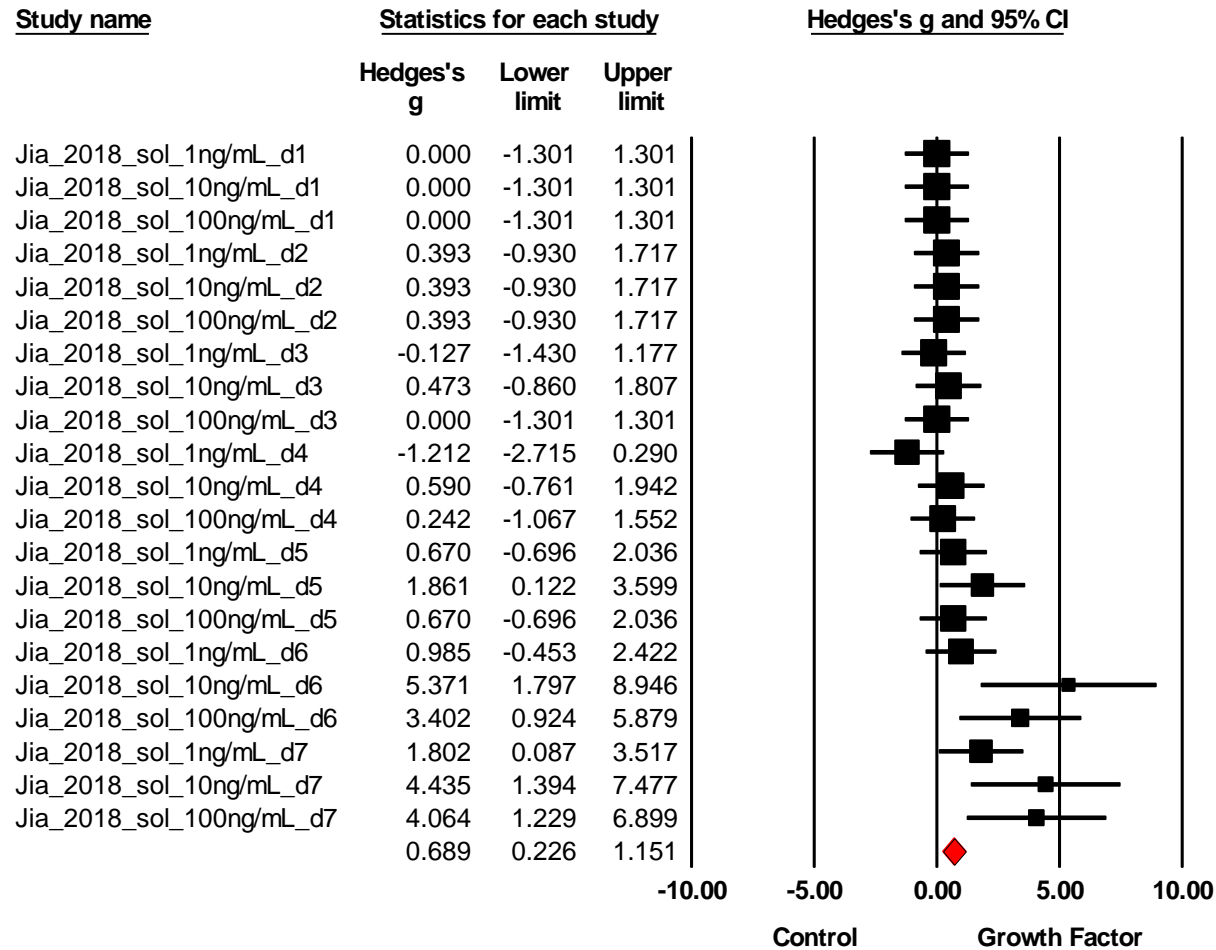

Figure S4: Forest plot displaying the effect of EGF on proliferation *in vitro*.

Supplement: Supplemental data [file Suppl_FigS4.pdf]
