# Supplementary material for: The Effect of Growth Factors on Vaginal Wound Healing: A Systematic Review and Meta-analysis
Source: Tissue Eng Part B Rev. 2023 Aug 8;29(4):429–40. doi: 10.1089/ten.teb.2022.0225 (PMC10701546; doi:10.1089/ten.teb.2022.0225)
Supplement: Supplemental data [file Suppl_FigS3.pdf]

Group by Time	Study name	Hedges's g	Lower limit	Upper limit
middle	Wu_2021_transfected_d9_c	4.334	1.892	6.776
middle	Wu_2021_transfected_d11_c	15.576	7.849	23.303
middle		5.958	2.902	9.014
short	Jia_2018_sol_1ng/mL_d1	0.000	-1.301	1.301
short	Jia_2018_sol_10ng/mL_d1	0.000	-1.301	1.301
short	Jia_2018_sol_100ng/mL_d1	0.000	-1.301	1.301
short	Jia_2018_sol_1ng/mL_d2	0.077	-1.225	1.380
short	Jia_2018_sol_10ng/mL_d2	0.077	-1.225	1.380
short	Jia_2018_sol_100ng/mL_d2	0.149	-1.155	1.454
short	Jia_2018_sol_1ng/mL_d3	0.321	-0.995	1.637
short	Jia_2018_sol_10ng/mL_d3	0.508	-0.831	1.847
short	Jia_2018_sol_100ng/mL_d3	0.321	-0.995	1.637
short	Jia_2018_sol_1ng/mL_d4	0.780	-0.608	2.168
short	Jia_2018_sol_10ng/mL_d4	1.516	-0.089	3.121
short	Jia_2018_sol_100ng/mL_d4	1.174	-0.317	2.665
short	Jia_2018_sol_1ng/mL_d5	1.724	0.040	3.407
short	Jia_2018_sol_10ng/mL_d5	2.888	0.675	5.102
short	Jia_2018_sol_100ng/mL_d5	2.063	0.239	3.887
short	Jia_2018_sol_1ng/mL_d6	1.606	-0.032	3.244
short	Jia_2018_sol_10ng/mL_d6	5.314	1.773	8.856
short	Jia_2018_sol_100ng/mL_d6	2.187	0.308	4.066
short	Jia_2018_sol_1ng/mL_d7	1.819	0.097	3.541
short	Jia_2018_sol_10ng/mL_d7	5.898	2.018	9.777
short	Jia_2018_sol_100ng/mL_d7	2.344	0.394	4.294
short	Wu_2021_transfected_d0_a	-0.451	-1.676	0.775
short	Wu_2021_transfected_d1_a	0.281	-0.932	1.494
short	Wu_2021_transfected_d2_a	0.946	-0.345	2.237
short	Wu_2021_transfected_d3_a	0.994	-0.306	2.293
short	Wu_2021_transfected_d4_a	3.051	1.131	4.971
short	Wu_2021_transfected_d5_a	4.018	1.710	6.326
short	Wu_2021_transfected_d6_a	4.111	1.764	6.459
short	Wu_2021_transfected_d0_b	0.304	-0.910	1.518
short	Wu_2021_transfected_d1_b	1.163	-0.170	2.496
short	Wu_2021_transfected_d2_b	3.889	1.634	6.143
short	Wu_2021_transfected_d3_b	1.738	0.263	3.214
short	Wu_2021_transfected_d4_b	3.380	1.332	5.428
short	Wu_2021_transfected_d5_b	5.469	2.531	8.408
short	Wu_2021_transfected_d6_b	7.661	3.719	11.604
short	Wu_2021_transfected_d0_c	-0.118	-1.325	1.088
short	Wu_2021_transfected_d1_c	-0.995	-2.295	0.305
short	Wu_2021_transfected_d3_c	4.317	1.883	6.752
short	Wu_2021_transfected_d5_c	1.676	0.218	3.135
short	Wu_2021_transfected_d7_c	3.309	1.289	5.330
short	Zhang_2016_mesh_d1	0.889	-0.487	2.264
short	Zhang_2016_mesh_d3	1.301	-0.176	2.777
short	Zhang_2016_mesh_d5	2.075	0.338	3.812
short		1.430	1.003	1.858
Overall		1.517	1.094	1.941

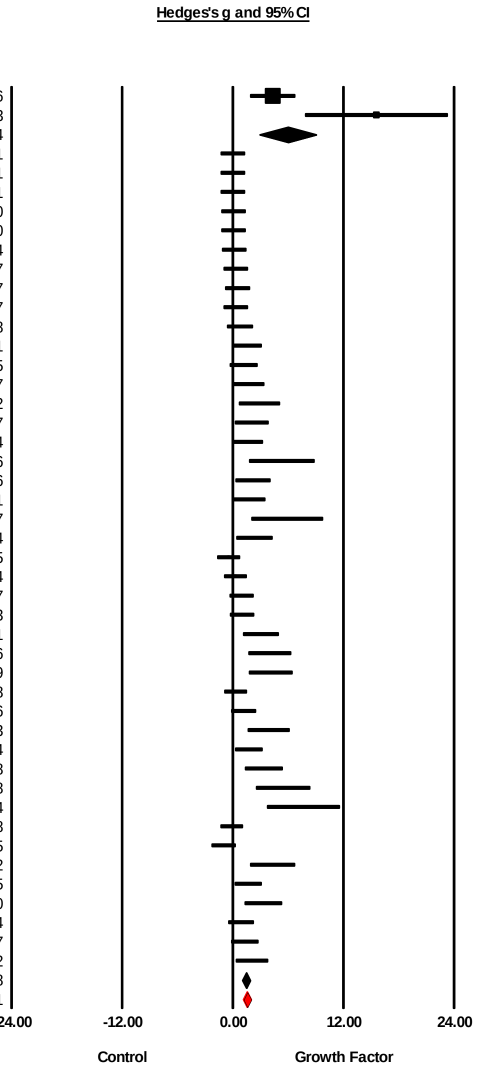

Figure S3: Subgroup analysis (short-term outcome assessment) regarding the effect of bFGF on proliferation *in vitro*.
