# Supplementary material for: The Effect of Growth Factors on Vaginal Wound Healing: A Systematic Review and Meta-analysis
Source: Tissue Eng Part B Rev. 2023 Aug 8;29(4):429–40. doi: 10.1089/ten.teb.2022.0225 (PMC10701546; doi:10.1089/ten.teb.2022.0225)
Supplement: Supplemental data [file Suppl_FigS5.pdf]

Study name	Statistics for each study		
	Hedges's g	Lower limit	Upper limit
Wu_2021_sol_d14	2.231	0.332	4.130
Wu_2021_sol_d28	5.205	1.726	8.684
Wu_2021_transfected_d14	2.899	0.680	5.117
Wu_2021_transfected_d28	12.388	4.601	20.176
	3.961	1.537	6.385

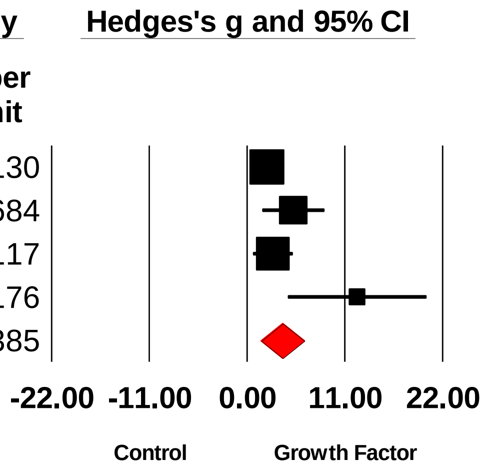

Figure S5: Forest plot displaying the effect of bFGF on (stem) cell differentiation into fibroblasts *in vitro*.
