# Supplementary material for: The Effect of Growth Factors on Vaginal Wound Healing: A Systematic Review and Meta-analysis
Source: Tissue Eng Part B Rev. 2023 Aug 8;29(4):429–40. doi: 10.1089/ten.teb.2022.0225 (PMC10701546; doi:10.1089/ten.teb.2022.0225)
Supplement: Supplemental data [file Suppl_FigS7.pdf]

A

Study name	Hedges's g	Lower limit	Upper limit
Su_2014_sol_10ng/mL_d14	1.121	-0.166	2.408
Su_2014_sol_50ng/mL_d14	3.236	1.467	5.006
Su_2014_sol_100ng/mL_d14	2.993	1.294	4.693
Su_2014_sol_10ng/mL_d28	0.632	-0.601	1.864
Su_2014_sol_50ng/mL_d28	2.360	0.828	3.892
Su_2014_sol_100ng/mL_d28	12.782	7.528	18.035
Su_2014_mesh_100ng/mL_d14	12.741	7.538	17.945
Su_2014_mesh_100ng/mL_d28	16.280	9.683	22.876
	4.737	2.612	6.863

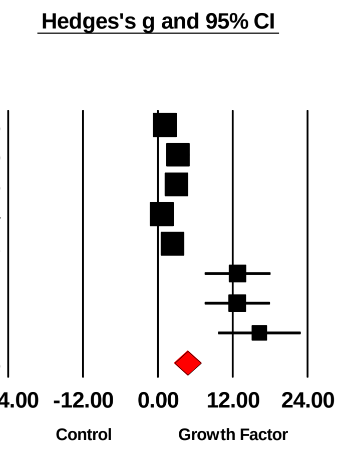

B

Study name	Hedges's g	Lower limit	Upper limit
Su_2014_sol_10ng/mL_d14	0.493	-0.729	1.716
Su_2014_sol_50ng/mL_d14	0.974	-0.293	2.242
Su_2014_sol_100ng/mL_d14	3.846	1.891	5.801
Su_2014_sol_10ng/mL_d28	0.118	-1.089	1.325
Su_2014_sol_50ng/mL_d28	0.301	-0.911	1.513
Su_2014_sol_100ng/mL_d28	5.080	2.717	7.443
Su_2014_mesh_100ng/mL_d14	1.960	0.654	3.266
Su_2014_mesh_100ng/mL_d28	2.777	1.252	4.301
	1.724	0.693	2.755

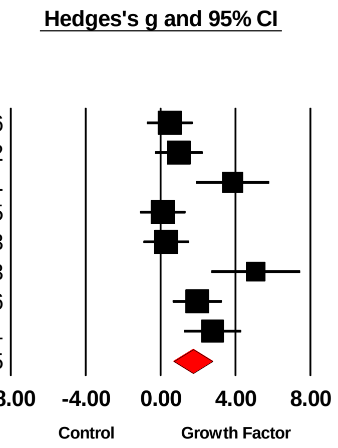

C

Study name	Hedges's g	Lower limit	Upper limit
Su_2014_sol_10ng/mL_d28	0.118	-1.089	1.325
Su_2014_sol_50ng/mL_d28	0.301	-0.911	1.513
Su_2014_sol_100ng/mL_d28	5.080	2.717	7.443
Su_2014_mesh_100ng/mL_d28	2.777	1.252	4.301
	1.866	-0.037	3.769

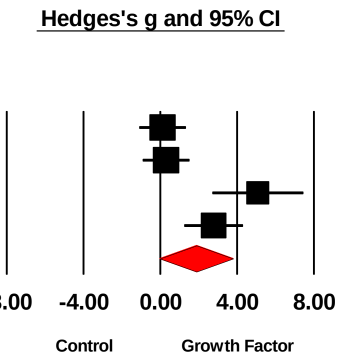

Figure S7: Forest plot displaying the effect of CTGF on the protein expression of Tenascin-C (A) and collagen type I (B and C). (C) Sensitivity analysis in which only the last reported outcome was analyzed.
