# Supplementary material for: The Effect of Growth Factors on Vaginal Wound Healing: A Systematic Review and Meta-analysis
Source: Tissue Eng Part B Rev. 2023 Aug 8;29(4):429–40. doi: 10.1089/ten.teb.2022.0225 (PMC10701546; doi:10.1089/ten.teb.2022.0225)
Supplement: Supplemental data [file Suppl_FigS8.pdf]

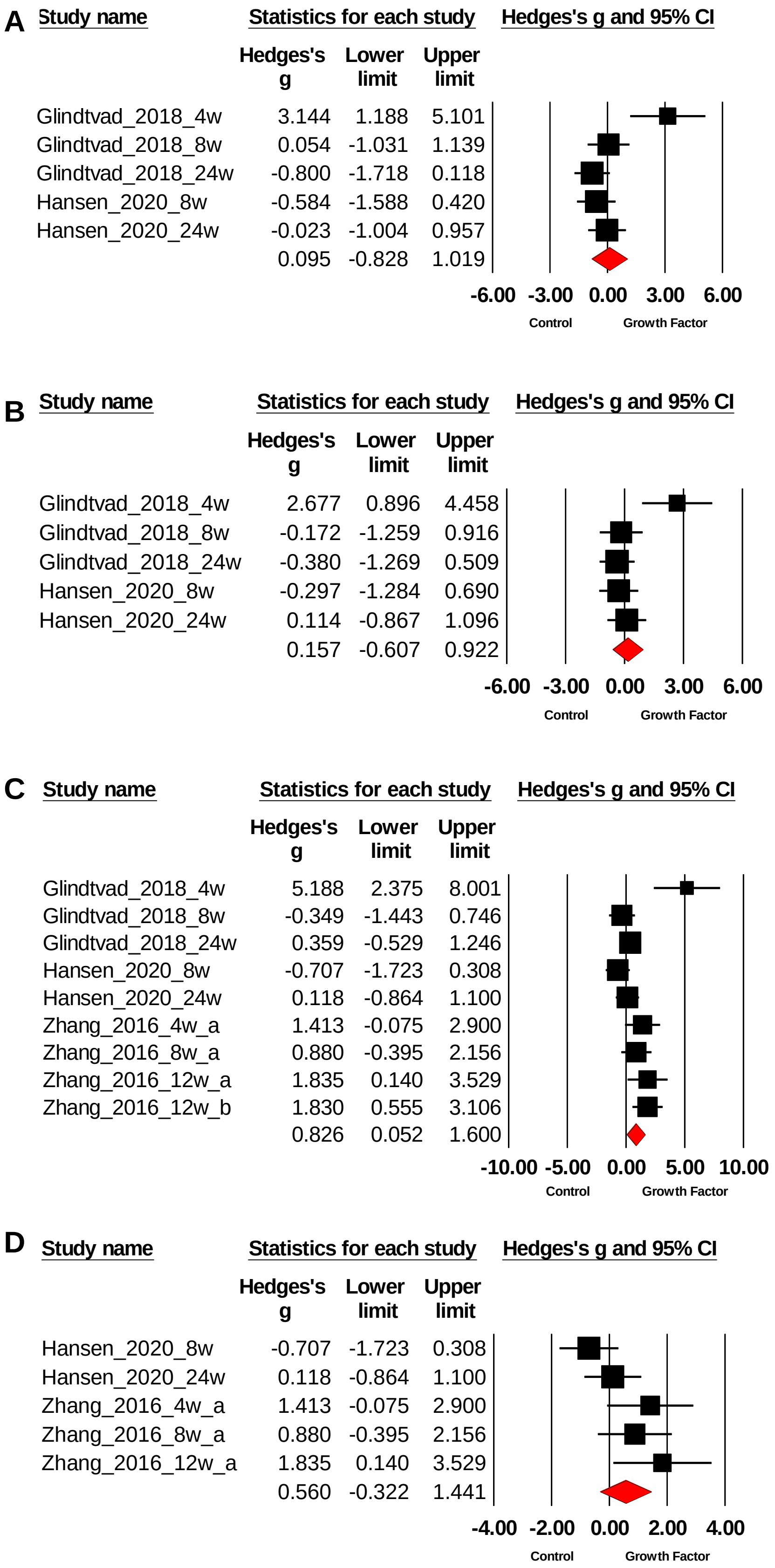

Figure S8: Forest plots displaying the effect of bFGF on gene expression of collagen type I (A) and collagen type III (B) and total collagen production (C-D) *in vivo*. (D) Sensitivity analysis in which only the histological comparisons were analyzed.
