# Supplementary material for: The Effect of Growth Factors on Vaginal Wound Healing: A Systematic Review and Meta-analysis
Source: Tissue Eng Part B Rev. 2023 Aug 8;29(4):429–40. doi: 10.1089/ten.teb.2022.0225 (PMC10701546; doi:10.1089/ten.teb.2022.0225)
Supplement: Supplemental data [file Suppl_FigS9.pdf]

A

Study name	Hedges's g	Lower limit	Upper limit
Glindtvad_2018_4w	1.788	0.298	3.277
Glindtvad_2018_8w	0.680	-0.442	1.802
Glindtvad_2018_24w	-0.194	-1.076	0.688
Hansen_2020_8w	0.064	-0.917	1.045
Hansen_2020_24w	-0.298	-1.285	0.689
Zhang_2016_4w	-2.428	-4.449	-0.407
Zhang_2016_8w	-1.600	-3.176	-0.024
Zhang_2016_12w	-1.233	-2.642	0.175
	-0.275	-0.996	0.446

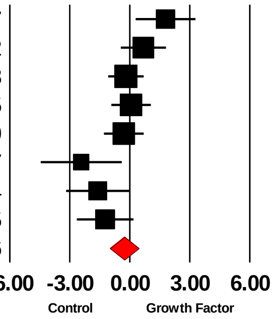

B

Study name	Hedges's g	Lower limit	Upper limit
Glindtvad_2018_4w	1.271	-0.085	2.628
Glindtvad_2018_8w	1.110	-0.070	2.290
Glindtvad_2018_24w	-0.201	-1.083	0.682
Hansen_2020_8w	0.159	-0.824	1.141
Hansen_2020_24w	-0.592	-1.597	0.413
Zhang_2016_4w	0.712	-0.512	1.936
Zhang_2016_8w	1.004	-0.314	2.323
Zhang_2016_12w	1.250	-0.166	2.666
	0.468	-0.042	0.979

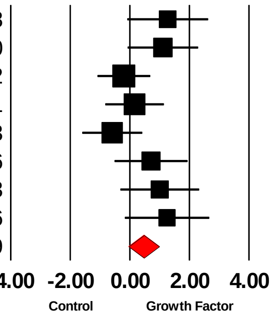

C

Study name	Hedges's g	Lower limit	Upper limit
Hansen_2020_8w	-0.147	-1.130	0.835
Hansen_2020_24w	-0.378	-1.369	0.613
Zhang_2016_4w	0.655	-0.553	1.864
Zhang_2016_8w	0.893	-0.386	2.173
Zhang_2016_12w	0.731	-0.498	1.961
	0.239	-0.271	0.749

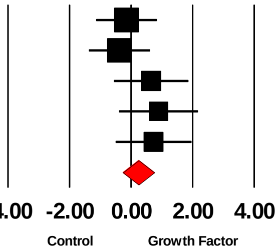

D

Study name	Hedges's g	Lower limit	Upper limit
Glindtvad_2018_4w	-4.456	-6.950	-1.962
Glindtvad_2018_8w	0.011	-1.074	1.096
Glindtvad_2018_24w	-0.436	-1.327	0.456
Hansen_2020_8w	-0.078	-1.059	0.903
Hansen_2020_24w	1.335	0.237	2.434
	-0.347	-1.473	0.780

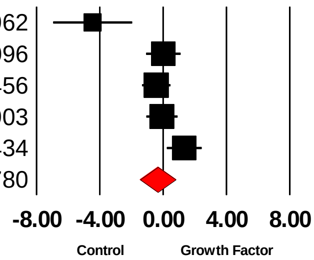

Figure S9: Forest plots displaying the effect of bFGF on biomechanical properties *in vivo*: (A) elastic modulus, (B) UTS, (C) strain at UTS and (D) explant thickness.
