# Supplementary material for: The Effect of Growth Factors on Vaginal Wound Healing: A Systematic Review and Meta-analysis
Source: Tissue Eng Part B Rev. 2023 Aug 8;29(4):429–40. doi: 10.1089/ten.teb.2022.0225 (PMC10701546; doi:10.1089/ten.teb.2022.0225)
Supplement: Supplemental data [file Suppl_FigS6.pdf]

A

Study name	Hedges's g	Lower limit	Upper limit
Wu_2021_sol_d14_a	3.281	0.867	5.696
Wu_2021_sol_d14_b	8.734	3.167	14.302
Wu_2021_sol_d28_a	6.899	2.429	11.369
Wu_2021_transfected_d7_c	1.295	-0.180	2.770
Wu_2021_transfected_d14_a	5.729	1.947	9.511
Wu_2021_transfected_d14_b	9.408	3.433	15.382
Wu_2021_transfected_d28_a	11.979	4.441	19.517
	5.770	3.026	8.515

Hedges's g and 95% CI

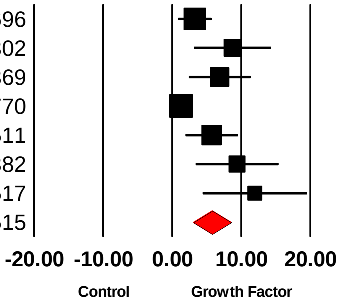

B

Study name	Hedges's g	Lower limit	Upper limit
Wu_2021_sol_d14_a	3.055	0.758	5.353
Wu_2021_sol_d14_b	6.191	2.139	10.242
Wu_2021_sol_d28_a	1.148	-0.335	2.631
Wu_2021_transfected_d7_c	2.491	0.587	4.395
Wu_2021_transfected_d14_a	8.690	3.149	14.231
Wu_2021_transfected_d14_b	16.594	6.227	26.961
Wu_2021_transfected_d28_a	5.525	1.862	9.189
	4.273	2.154	6.393

Hedges's g and 95% CI

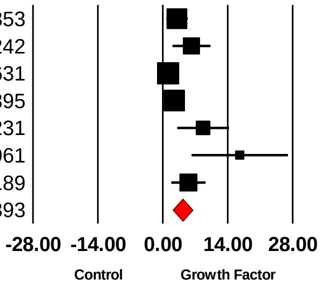

Figure S6: Forest plot displaying the effect of bFGF on the protein expression of collagen type I (A) and collagen type III (B).
